# Synthesis, In Vitro Evaluation and Molecular Docking of the 5-Acetyl-2-aryl-6-hydroxybenzo[*b*]furans against Multiple Targets Linked to Type 2 Diabetes

**DOI:** 10.3390/biom10030418

**Published:** 2020-03-07

**Authors:** Malose J. Mphahlele, Yee Siew Choong, Marole M. Maluleka, Samantha Gildenhuys

**Affiliations:** 1 Department of Chemistry, University of South Africa, Private Bag X06, Florida 1710, South Africa; 2 Institute for Research in Molecular Medicine (INFORMM), Universiti Sains Malaysia, Penang 11800, Malaysia; yeesiew@usm.my; 3 Department of Life & Consumer Sciences, College of Agriculture and Environmental Sciences, University of South Africa, Private Bag X06, Florida 1710, South Africa; gildes@unisa.ac.za

**Keywords:** 5-acetyl-2-aryl-6-hydroxybenzo[*b*]furans, α-glucosidase, protein tyrosine phosphatase 1B, β-secretase, antioxidant activity, cyclooxygenase-2, molecular docking

## Abstract

The 5-acetyl-2-aryl-6-hydroxybenzo[*b*]furans **2a**–**h** have been evaluated through in vitro enzymatic assay against targets which are linked to type 2 diabetes (T2D), namely, α-glucosidase, protein tyrosine phosphatase 1B (PTP1B) and β-secretase. These compounds have also been evaluated for antioxidant activity using the 2,2-diphenyl-1-picrylhydrazyl (DPPH) free-radical scavenging method. The most active compounds against α-glucosidase and/or PTP1B, namely, 4-fluorophenyl **2c**, 4-methoxyphenyl **2g** and 3,5-dimethoxyphenyl substituted **2h** derivatives were also evaluated for potential anti-inflammatory properties against cyclooxygenase-2 activity. The Lineweaver-Burk and Dixon plots were used to determine the type of inhibition on compounds **2c** and **2h** against α-glucosidase and PTP1B receptors. The interactions were investigated in modelled complexes against α-glucosidase and PTP1B via molecular docking.

## 1. Introduction

Type 2 diabetes (T2D) is a chronic metabolic disorder that has reached epidemic proportion throughout the world and this disease is characterized by a defect in the secretion of insulin and resistance to insulin in its target organs [1]. The deficiency is attributed to increased glucose levels in blood also known as post-prandial hyperglycaemia (PPHG), which has emerged as a prominent and early deficiency in T2D [2]. Several cellular and metabolic targets are involved to ameliorate T2D complications, such as, α-glucosidase and protein tyrosine phosphatase 1 beta (PTP1B) inhibition, correction of abnormal insulin signalling, promotion of the action of insulin on its target tissues, including antioxidative and anti-inflammatory action [3]. α-Glucosidase, a membrane bound enzyme in small intestine plays a key role in inhibiting hydrolysis of carbohydrates. This enzyme acts on the 1,4-α glycosidic bonds by breaking down starch and disaccharides into single absorbable α-glucose molecules [4]. Inhibitors of α-glucosidase act in a competitive or non-competitive manner to delay the absorption of carbohydrates by the small intestine, thus prolonging the overall carbohydrate digestion time ultimately lowering post-prandial blood glucose and insulin levels [5]. PTP1B, on the other hand, is an intracellular non-receptor protein tyrosine phosphatase, which is highly expressed in insulin targeted tissues such as liver and muscle including fats [6] and has also received much attention due to its role in type 2 diabetes mellitus and obesity [7]. PTP1B works specifically by dephosphorylating the residues of phosphotyrosine both from the insulin receptor (IR) and the insulin receptor substrate [8]. α-Glucosidase and PTP1B are the key targets to treat diabetes and obesity [9] and compounds with dual inhibitory activity against these enzymes exhibit synergistic effects to prevent hyperglycaemia, in turn, effectively improve insulin sensitization [10]. In vivo studies have demonstrated that β-secretase (BACE-1) regulates the amount of IR and insulin signalling in the liver making inhibitors of this enzyme, to enhance insulin signalling during diabetes [11,12]. Likewise, oxidative stress is implicated in the early stages and progression of T2D due to disruption of the pro-oxidant/antioxidant balance leading to the development of insulin resistance [13,14]. Inflammatory responses, on the other hand, contribute to T2D occurrence by causing insulin resistance, and, in turn, they are intensified in the presence of hyperglycaemia and promote long-term diabetic complications [15]. It is envisaged that targeting the key enzymes associated with pathogenesis and progression of T2D and oxidative stress as well as inflammatory pathways could be a significant strategy for treating this multifunctional disease.

Phenols and hydroxyacetophenones continue to attract considerable attention in medicinal chemistry especially in the context of drug development. Their heterocyclic derivatives such as benzofurans, for example, are endowed with several pharmacological properties including antihyperglycemic, antidiabetic, anti-Alzheimer’s, anti-inflammatory, anti-oxidant and antitumor activities and some derivatives serve as enzyme inhibitors [12,16,17,18]. The naturally occurring 2,4-dihydroxy-5-methylacetophenone and its derivatives have been found to exhibit α-glucosidase inhibitory properties [19]. Likewise, the 2-acylbenzofurans were found to inhibit α-glucosidase and to exhibit hypoglycaemic activity [20]. The analogous 2-unsubstituted 5-acetyl-4-hydroxybenzofurans and 5-acetyl-6-hydroxybenzofurans as well as their benzoylated derivatives, on the other hand, exhibit an inhibitory effect against PTP1B activity [21]. Several naturally occurring 2-arylbenzofurans (sanggenofuran A, mulberrofuran D2, mulberrofuran D, morusalfuran B and mulberrofuran H), isolated from the root bark of *Morus alba,* have also been found to exhibit an inhibitory effect against PTP1B activity [22]. Based on the capability of *ortho-*hydroxyacetophenones to target α-glucosidase and the PTP1B inhibitory properties of *ortho-*hydroxyacetylbenzofuran derivatives and the 2-arylbenzofuran analogues, we decided to synthesize the *ortho-*hydroxyacetyl substituted 2-arylbenzofurans. The main aim was to evaluate these compounds through enzymatic assays in vitro for potential dual inhibitory properties against α-glucosidase and PTP1B activities. Due to the multifunctional origin of diabetes and contributing factors to this disease, the spectrum of targets was extended to include β-secretase, free radical scavenging and anti-inflammatory potential of these compounds. Their possible mode of interaction with α-glucosidase or PTP1B was investigated through kinetic and molecular docking studies.

## 2. Materials and Methods

### 2.1. General

The melting point (mp.) values of the prepared compounds were recorded on a Thermocouple digital melting point apparatus (Mettler Toledo LLC, Columbus, OH, USA) and are uncorrected. Their infrared (IR) spectra were recorded using the thin-film method on a Bruker VERTEX 70 FT-IR Spectrometer (Bruker Optics, Billerica, MA, USA) equipped with a diamond attenuated total reflectance (ATR) accessory. The Merck kieselgel 60 (0.063–0.200 mm) (Merck KGaA, Frankfurt, Germany) was used as a stationary phase for column chromatography. The proton (^1^H-) and carbon-13 (^13^C-) nuclear magnetic resonance (NMR) spectra of the prepared compounds were obtained as CDCl_3_ or dimethyl sulfoxide-*d*_6_ (DMSO-*d*_6_) solutions using Agilent 500 MHz NMR spectrometer (Agilent Technologies, Oxford, UK). The chemical shifts are quoted relative to the tetramethylsilane (TMS) peak as an internal reference standard. The high-resolution mass spectra were recorded at the University of Stellenbosch using a Waters Synapt G2 Quadrupole Time-of-flight mass spectrometer (Waters Corp., Milford, MA, USA) at an ionization potential of 70 eV. The synthesis and analytical data for 2,4-dihydroxy-5-iodoacetophenone, were reported in our previous investigation [23].

### 2.2. Typical Procedure for the Synthesis of Compounds **2a**–**h**

To a three necked-flask equipped with a condenser and rubber septa, 2,4-dihydroxy-5-iodoacetophenone (1.00 g, 3.60 mmol), PdCl_2_(PPh_3_)_2_ (0.13 g, 0.18 mmol), CuI (0.07 g, 0.37 mmol), PPh_3_ (0.096 g, 0.37 mmol) and K_2_CO_3_ (0.60 g, 4.34 mmol) were added followed by 9:1 DMF-water (*v*/*v*, 50 mL). The mixture was purged for 30 min with an argon gas. A solution of phenylacetylene derivative (1.2 equivalent) in DMF (1 mL) was introduced to the mixture using a syringe. The stirred mixture was heated at 80 °C for 3 h under an argon atmosphere and the mixture was poured into an ice-cold water. The product was extracted with chloroform and the combined organic layers were dried over anhydrous magnesium sulphate. The salt was filtered off and the solvent was evaporated under reduced pressure on a rotary evaporator. The residue was purified by silica gel column chromatography using toluene as an eluent to afford compound **2** as a solid. The following compounds were prepared in this fashion.

#### 2.2.1. 1-[6-Hydroxy-2-phenylbenzofuran-5-yl]ethanone (**2a**)

Yellow solid (0.38 g, 84%), mp. 172‒173 °C; ν_max_ (ATR) 3083 (OH), 1640 (C=O), 1611, 1446, 1366, 1288, 1247, 1140, 1012, 799, 765 cm^-1^; ^1^H-NMR (500 MHz, CDCl_3_) 12.52 (1H, br s, OH), 7.96 (1H, s, H-4), 7.85 (2H, d, *J* = 8.0 Hz, Ar), 7.48 (1H, t, *J* = 7.5 Hz, Ar), 7.42 (2H, d, *J* = 7.0 Hz, Ar), 7.06 (1H, s, H-7) 6.94 (1H, s, H-3), 2.69 (3H, s, -CH_3_); ^13^C-NMR (125 MHz, CDCl_3_) 204.0, 161.2, 159.6, 156.9, 129.7, 128.9, 128.8, 124.7, 123.5, 122.0, 117.0, 100.8, 96.6, 26.9; HRMS (ES^+^): *m*/*z* [M + H]^+^ calcd for C_16_H_11_O_3_: 251.0708; found 251.0820.

#### 2.2.2. 1-[2-(3-Fluorophenyl)-6-hydroxybenzofuran-5-yl]ethanone (**2b**)

Brown solid (0.33 g, 69%), mp. 165‒167 °C; ν_max_ (ATR) 3081 (OH), 1636 (C=O), 1618, 1573, 1487, 1446, 1370, 1331, 1270, 1154, 1141, 864,823, 780, 654 cm^-1^; ^1^H-NMR (500 MHz, CDCl_3_) 12.53 (1H, br s, OH), 7.96 (1H, s, H-4), 7.65 (1H, d, *J* = 8.5 Hz, Ar), 7.42 (2H, dd, *J* = 8.5 and 2.0 Hz, Ar), 7.14 (1H, t, *J* = 8.5 Hz, Ar), 6.95 (1H, s, H-3), 2.70 (3H, s, -CH_3_); ^13^C-NMR (125 MHz, CDCl_3_) 204.1, 163.0 (d, ^1^*J*_CF_ = 245.9 Hz), 161.5, 159.5, 155.5 (d, ^4^*J*_CF_ = 3.2 Hz), 131.7 (d, ^3^*J*_CF_ = 8.6 Hz), 130.5 (d, ^3^*J*_CF_ = 8.3 Hz), 123.8, 121.7, 120.4 (d, ^4^*J*_CF_ = 3.0 Hz), 117.2, 115.6 (d, ^2^*J*_CF_ = 21.3 Hz), 111.6 (d, ^2^*J*_CF_ = 23.7 Hz), 101.9, 96.7, 26.9; HRMS (ES^+^): *m*/*z* [M + H]^+^ calcd for C_16_H_10_FO_3_: 269.0614; found 269.0605.

#### 2.2.3. 1-[2-(4-Fluorophenyl)-6-hydroxybenzofuran-5-yl]ethanone (**2c**)

Brown solid (0.35 g, 73%), mp. 194‒195 °C; ν_max_ (ATR) 3082 (OH), 1626 (C=O), 1599, 1504, 1372, 1275, 1218, 1140, 844, 807, 795, 514 cm^-1^; ^1^H-NMR (500 MHz, CDCl_3_) 12.52 (1H, br s, OH), 7. 95 (1H, s, H-4), 7.79 (2H, dt, *J* = 8.7 Hz and, *J*_HF_ = 5.7 Hz, H-2′,6′), 7.14 (2H, dt, *J*_HH_ = 8.7 Hz and *J*_HF_ = 9.6 Hz, H-3′,5′), 7.05 (1H, s, H-7), 6.87 (1H, s, H-3), 2.70 (3H, s, –CH_3_); ^13^C-NMR (125 MHz, CDCl_3_) 204.0, 162.9 (d, ^1^*J*_CF_ = 249.6 Hz), 161.2, 159.5, 156.0, 126.6 (d, ^3^*J*_CF_ = 8.1 Hz), 126.0 (d, ^4^*J*_CF_ = 3.2 Hz), 123.4, 122.0, 117.1, 116.0 (d, ^2^*J*_CF_ = 22.0 Hz), 100.5, 99.7, 26.9; HRMS (ES^+^): m/z [M + H]^+^ calcd for C_16_H_10_FO_3_: 269.0614; found 269.0605.

#### 2.2.4. 1-[2-(3-Chlorophenyl)-6-hydroxybenzofuran-5-yl]ethanone (**2d**)

Yellow solid (0.36 g, 74%), mp.178–179 °C; ν_max_ (ATR) 3081 (OH), 1675 (C=O), 1587, 1561, 1474, 1359, 1275, 1144, 1078, 882, 788, 682,cm^-1^; ^1^H-NMR (500 MHz, CDCl_3_) 12.50 (1H, br s, OH), 7.99 (1H, s, H-4), 7.80 (1H, s, Ar), 7.70 (1H, d, *J* = 8.0 Hz, Ar), 7.38 (1H, d, *J* = 8.5 Hz, Ar), 7.32 (1H, d, *J* = 7.5 Hz, Ar), 7.07 (1H, s, H-7), 6.98 (1H, s, H-3), 2.72 (3H, s, CH_3_), ^13^C-NMR (125 MHz, CDCl_3_) 203.9, 161.5, 159.6, 155.3, 134.9, 131.4, 130.1, 129.7, 128.7, 124.7, 123.7, 122.7, 117.3, 102.0, 99.7, 29.6; HRMS (ES^+^): *m*/*z* [M + H]^+^ calcd for C_16_H_11_O_3_^35^Cl: 285.0318; found 285.0299.

#### 2.2.5. 1-[2-(4-Chlorophenyl)-6-hydroxybenzofuran-5-yl]ethanone (**2e**)

Yellow solid (0.43 g, 77%), mp. 170–173 °C; ν_max_ (ATR) 3082 (OH), 1676 (C=O), 1614, 1502, 1405, 1363, 1267, 1199, 1033, 987, 854, 746, 666 cm^−1^; ^1^H-NMR (500 MHz, CDCl_3_) 12.52 (1H, br s, OH), 7.96 (1H, s, H-4), 7.81 (2H, d, *J* = 8.0 Hz, H-2′,6′), 7.42 (2H, d, *J* = 8.0 Hz H-3′,5′), 7.06 (1H, s, H-7), 7.03 (1H, s, H-3), 2.92 (3H, s, CH_3_); ^13^C-NMR (125 MHz, CDCl_3_) 202.4, 156.4, 132.6, 130.2, 128.3, 128.0, 127.9, 127.7, 126.7, 123.0, 121.5, 116.6, 101.1, 39.8; HRMS (ES^+^): *m*/*z* [M + H]^+^ calcd for C_16_H_11_^35^ClO_3_: 285.0312; found 285.0318.

#### 2.2.6. 1-[6-Hydroxy-2-(4-tolyl)benzofuran-5-yl]ethanone (**2f**)

Yellow solid (0.41 g, 83%), mp. 169–172 °C; ν_max_ (ATR) 3082 (OH), 1676 (C=O), 1614, 1502, 1405, 1363, 1267, 1199, 1033, 987, 854, 746, 666 cm^−1^; ^1^H-NMR (500 MHz, CDCl_3_) 12.50 (1H, br s, OH), 7.95 (1H, s, H-4), 7.71 (2H, d, *J* = 8.0 Hz, Ar), 7.27 (2H, d, *J* = 8.0 Hz, Ar), 7.06 (1H, s, H-7), 6.88 (1H, s, H-3), 2.72 (3H, s, CH_3_), 2.41 (3H, s, CH_3_); ^13^C-NMR (125 MHz, CDCl_3_) 203.9, 161.2, 159.6, 138.9, 129.5, 127.0, 125.7, 124.7, 123.1, 122.2, 117.0, 100.0, 99.6, 26.8, 21.3; HRMS (ES^+^): *m*/*z* [M + H]^+^ calcd for C_17_H_14_O_3_: 265.0850; found 265.0865.

#### 2.2.7. 1-[6-Hydroxy-2-(4-methoxyphenyl)benzofuran-5-yl]ethanone (**2g**)

Yellow solid (0.39 g, 78%), mp. 197‒198 °C; ν_max_ (ATR) 3081 (OH), 1637 (C=O), 1609, 1505, 1369, 1274, 1252, 1164, 1140, 1015, 836, 815, 802, 659 cm^−1^; ^1^H-NMR (500 MHz, CDCl_3_) 12.51 (1H, br s, OH), 7. 90 (1H, s, H-4), 7.73 (2H, d, *J* = 9.0 Hz, Ar), 7.03 (1H, s, H-7), 6.97 (2H, d, *J* = 9.0 Hz, Ar;), 6.78 (1H, s, H-3),3.86 (3H, s, –OCH_3_), 2.69 (3H, s, –CH_3_); ^13^C-NMR (125 MHz, CDCl_3_) 204.0, 160.1, 161.0, 159.5, 157.0, 126.2, 122.9, 122.5, 122.4, 116.9, 114.3, 99.5, 99.1, 55.4, 26.9; HRMS (ES): found 282.0892. C_17_H_13_O_4_ requires: 282.0926.

#### 2.2.8. 1-[6-Hydroxy-2-(3,5-dimethoxyphenyl)benzofuran-5-yl]ethanone (**2h**)

Yellow solid (0.33 g, 69%), mp.155–156 °C; ν_max_ (ATR), 1676 (C=O), 3082 (OH) 1614, 1502, 1405, 1363, 1267, 1199, 1033, 987, 854, 746, 666 cm^−1^; ^1^H-NMR (500 MHz, CDCl_3_) 12.51 (1H, br s, OH), 7.95 (1H, s, H-4), 7.05 (1H, s, H-7), 6.96 (2H, s, Ar), 6.91 (1H, s, Ar), 6.48 (1H, s, H-3), 3.87 (6H, s, 2 × CH_3_), 2.70 (3H, s, CH_3_); ^13^C-NMR (125 MHz, CDCl_3_) 203.9, 161.3, 161.1 (2 × C), 159.5, 156.7, 131.4, 123.5 (2 × C), 121.9, 117.1, 102.9, 101.2 (2 × C), 99.6, 55.5 (2 × C), 29.6,; HRMS (ES^+^): *m*/*z* [M + H]^+^ calcd for C_18_H_16_O_5_: 311.0909; found 311.0919.

### 2.3. Bioassays

#### 2.3.1. Inhibition of α-Glucosidase Activity by Compounds by **2a**–**h**

α-Glucosidase type 1 from baker’s yeast, *p*-nitrophenyl-α-d-glucopyranoside (PNP-G) and acarbose were purchased from Sigma Aldrich (Pty) Ltd. (Modderfontein, Johannesburg, South Africa). The α-glucosidase inhibitory activity of compounds **2a**–**h** was evaluated in triplicates in a 96 well microplate following a protocol by Shi et al. [24]. In a 96-well plate, α-glucosidase (20 μL of enzyme solution of 0.48 U/mL in 100 mM phosphate buffer of pH 6.8) was incubated with different concentrations (0–100 μM) of the test compounds (**2a**–**h** and acarbose as positive control) in DMSO at 37 °C, for 10 min. Then 20 µL of 2 mM PNP-G was added and the absorbances were recorded at a wavelength of 405 nm using Varioskan flash microplate spectrophotometer (Thermo Scientific, Waltham, MA, USA). The IC_50_ values of these compounds were calculated through the nonlinear regression analysis using Origin Pro 9.0 (OriginLab Corporation, Northampton, MA, USA) and are expressed as the mean standard deviation (SD) of three distinct experiments.

#### 2.3.2. Inhibition PTP1B Activity by Compounds **2a**–**h**

The PTP1B inhibitory activity of compounds **2a**–**h** and sodium orthovanadate was evaluated in triplicates in a 96 well microplate following a protocol by Goldstein et al. [25]. Activity of PTPase (Sigma Aldrich, Modderfontein, South Africa) was evaluated using *p*-nitrophenylphosphate (PNPP) as the substrate. A 10 μL of PTP1B solution (0.02 U/mL) in 50 mM Hepes buffer (pH 7.0) with different concentrations (0.5, 10, 25, 50, 100 μM) of the test compounds and the reference standard were incubated at 25 °C for 10 min. After pre-incubation, 25 μL of HEPES buffer and 25 μL of 2 mM (pNPP) were added and the mixtures were incubated at 37 °C for 30 min. The reaction was stopped by the addition of 30 μL of 3 M NaOH and absorbance was determined at wavelength of 410 nm. Control tubes without the enzyme were ran in parallel to nullify the nonenzymatic reaction and for calculating the concentration of *p*-nitrophenolate ions produced in the reaction mixture. A molar extinction coefficient of 1.78 ×10^4^ M^-1^ cm^-1^ was used to calculate the concentration of *p*-nitrophenolate ions produced in the reaction mixture.

#### 2.3.3. In Vitro β-Secretase (BACE-1) Enzyme Assay

β-Secretase inhibitory activities of compounds **2a**–**h** were evaluated by a fluorescence resonance energy transfer (FRET) assay (Pan Vera) with a recombinant baculovirus-expressed β-secretase and a specific substrate (Rh-EVNLDAEFK-Quencher), according to the manufacturer’s protocol (Thermo Fisher Scientific Corporation, Johannesburg, Gauteng, South Africa). A mixture of human recombinant β-secretase (1.0 U/mL), the substrate (75 µM in 50 mM ammonium bicarbonate) and the test compound (various concentrations of **2a**–**h** and quercetin in DMSO) dissolved in an assay buffer (50 mM sodium acetate, pH 4.5) was incubated in a 96 well plate for 60 min room temperature. The assays were conducted in triplicates. The increase in fluorescence intensity produced by the substrate was measured on a fluorescence microplate reader with an excitation wavelength of 545 nm and an emission wavelength of 590 nm. The percentage inhibition was calculated using the following equation:

$$\mathrm{Inhibition}\;\left(\%\right)=\frac{1–\left(S\;–S_{0}\right)}{\left(C\;–C_{0}\right)}\;\times 100,$$

where C is the fluorescence of control (enzyme, assay buffer and substrate) after 60 min of incubation, C_0_ is the fluorescence of control at time 0, S is the fluorescence of tested samples (enzyme, sample solution and substrate) after 60 min. of incubation and S_0_ the fluorescence of the tested samples at time 0. The IC_50_ values were calculated with Origin Pro 9.0. (OriginLab Corporation).

#### 2.3.4. DPPH Radical Scavenging Activity of Compounds 2a–h

Antioxidant activities of compounds **2a**–**h** against ascorbic acid (Sigma Aldrich, Saint Louis, Missouri, USA) as a positive control were evaluated using 2,2-diphenyl-1-picrylhydrazyl (DPPH) radical scavenging assay following a reported method by Zhu et al. [26]. Triplicate solutions of the test compounds and the control in methanol (concentrations ranging from 0 µM to 50 µM) were treated with a solution of DPPH (0.3 mM) in methanol. The mixtures were incubated in the dark for 45 min and the absorbances were recorded at the wavelength of 512 nm using Varioskan flash microplate spectrophotometer reader. The inhibition was calculated in terms of percentage using the formula below,

$$\mathrm{DPPH}\;\mathrm{radical}\;\mathrm{scavenged}\;\left(\%\right)=\frac{\mathrm{AbC}\;-\;\mathrm{AbS}}{\mathrm{AbC}}\times 100,$$

where AbC is absorbance of control and AbS the absorbance of the test sample. A graph of percentage inhibition of free radical activity was plotted against concentration of the sample. The IC_50_ value, that is, concentration of the compound required to reduce the absorbance of the DPPH control solution by 50% was obtained from the graph.

#### 2.3.5. COX-2 Inhibitory Assays of **2c**, **2g** and **2h**

Solutions of compounds **2c**, **2g**, **2h** and the reference standard drug—celecoxib—in DMSO at concentrations (0-100 μM) were evaluated in vitro for inhibitory activity against cyclooxygenase-2 (COX-2) using a COX-2 inhibitor screening kit (cat no. K547-100) following the manufacturer’s procedure (Biovision Inc, Milpitas, CA, USA). The absorbance was measured using a Varioskan flash microplate spectrophotometer and the IC_50_ values were calculated with the aid of Origin Pro 9.0. (OriginLab Corporation).

### 2.4. Kinetic Studies of **2c** and **2h** towards α-Glucosidase and PTP1B Inhibition

#### 2.4.1. Kinetic Studies of **2c** and **2h** towards α-Glucosidase Inhibition

The kinetics studies on compounds **2c** and **2h** were performed according to the reaction conditions in 2.3.1 with inhibitors of various concentrations (0.5, 5, 10, 20 µM) and the ranges of final substrate concentration were 0–25 µM. The type of inhibition was determined by using Lineweave-Burk plot (the inverse of velocity (1/*v*) against the inverse of the substrate concentration (1/[S]). The inhibitor constant was obtained by Dixon plot (the inverse of velocity (1/*v*) against concentration of inhibitor at each substrate concentration).

#### 2.4.2. Kinetic Studies of **2c** and **2h** towards PTP1B Inhibition

Compounds **2c** and **2h** were subjected to kinetic analysis following the procedure outlined in 2.3.2 with inhibitors of various concentrations (0.5, 5, 10, 20 µM). The assay was performed with substrate of various concentrations (0, 2.5, 6.25, 12.5, 25.0 µM). The absorbance due to the *p*-nitrophenol produced by dephosphorylation of PTP1B was measured at 405 nm. The kinetic parameters were determined using Lineweaver-Burk double-reciprocal plots and the Dixon the Dixon plot method at increasing substrate and compound concentrations.

### 2.5. Molecular Docking Studies into α-Glucosidase and PTP1B Active Sites

#### 2.5.1. Protein Structure

The primary sequence of *Saccharomyces* *cerevisiae* α-glucosidase (584 amino acids) was obtained from UniProtKB [27] with accession number P53341. The secondary structure prediction was performed using JPred [28], PORTER [29], Predict Protein [30], PSIPRED [31], SCRATCH [32] and YASPIN [33]. Templates for comparative modelling using MODELLERv9.20 [34] was identified from BLAST search against RCSB Protein Data Bank. A total of 200 comparative models were built and the model from the best DOPE fold assessment score [35] was selected for docking simulation after being evaluated via Verify_3D [36] and ProCheck Ramachandran plot [37]. On the other hand, PTP1B in complex with catalytic inhibitor was obtained from PDB (ID 1NNY; 2.40 Å) [38] while PTB1B in complex with allosteric inhibitor was obtained from PDB (ID 1T49; 1.90 Å) [39]. All heteroatoms and water molecules were removed. Polar hydrogen atoms, Kollman-Amber united atom partial charges and solvation parameters were added by utilizing AutoDockTool [40].

#### 2.5.2. Ligand Structure

The ligands in α-glucosidase and PTB1B were used as the control docking. The initial coordinates for test compound **2a**–**2h** were generated using Avogadro [41]. All ligands were retained with polar hydrogen atoms. Gasteiger charges and torsional angles were added by utilizing AutoDockTools.

#### 2.5.3. Molecular Docking Simulation

The grid box was centred at the protein binding site (40 × 40 × 40 points with 0.375 Å grid spacing). AutoDock4.2.6 [40] was used to perform 100 docking runs for each ligand. Together with Lamarckian genetic algorithm [40], 2,500,000 energy evaluations and maximum 27,000 generation were employed. The population size was set at 150 with 0.02 and 0.8 mutation rate and crossover rate, respectively. The ligand conformation in the most populated cluster with most negative binding free energy was selected for further interaction analysis.

## 3. Results and Discussion

### 3.1. Chemistry

**Scheme 1:**
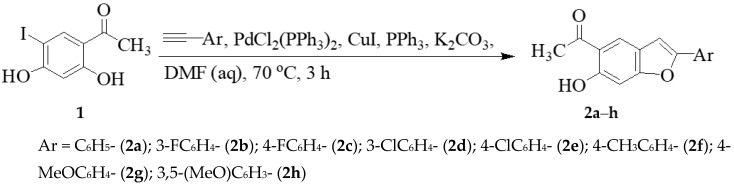
One-pot tandem Sonogashira cross-coupling and Cacchi-type annulation of **1**.

The above-mentioned compounds were accessible via tandem Sonogashira cross-coupling and Cacchi-type heteroannulation of the known 2,4-dihydroxy-5-iodoacetophenone (**1**) [23]. The latter was subjected to phenylacetylene derivatives in the presence of dichlorobis(triphenylphosphine)palladium(II)-copper iodide catalyst mixture, triphenylphosphine as ligand and potassium carbonate as a base in aqueous dimethylformamide at 70 °C (Scheme 1). We isolated in each case after 3 h by aqueous work-up and purification by column chromatography on silica gel the homo-coupled dimer and the cross-coupled product in sequence. The cross-coupled products were characterized using a combination of NMR (^1^H- and ^13^C-), IR and mass spectrometric techniques as the *ortho-*hydroxyacetyl substituted 2-arylbenzofurans **2a**–**h**. The ^1^H-NMR and ^13^C-NMR spectra of these compounds obtained as CDCl_3_ solutions (Figure S1 in the Supplementary Information, SI) revealed the presence of an intense singlet around δ 2.70 ppm for the methyl group and increased number of signals in the aromatic region as well as a broad singlet for the hydroxyl proton around δ 12.52 ppm. No deuterium exchange occurred in CDCl_3_–D_2_O mixture presumably due to strong intramolecular hydrogen bonding of this hydroxyl proton with the carbonyl oxygen. The ^13^C-NMR spectra of these compounds, on the other hand, lacked a set of singlets in the region δ 80–100 ppm typical for the acetylene moiety, which confirmed cycloisomerization.

**Figure 1:**
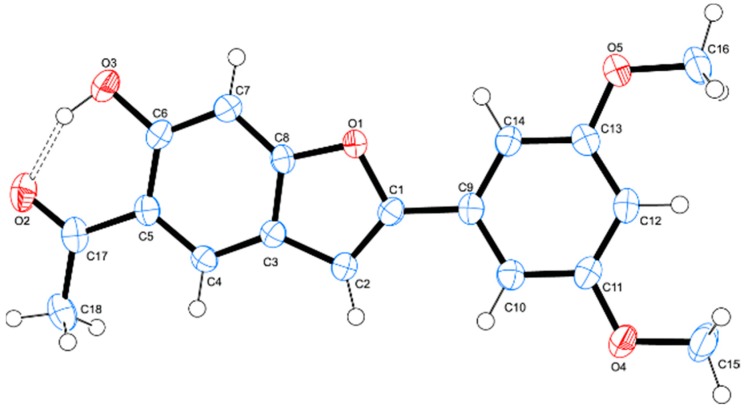
Oak Ridge Thermal Ellipsoid Plot (ORTEP) diagram (50% probability level) of **2h** showing atom numbering scheme.

We were able to obtain single crystals of the 3,5-dimethoxyphenyl substituted derivative **2h** suitable for X-ray diffraction analysis by slow evaporation of ethanol. CCDC 1975570 contains the supplementary crystallographic data of **2h**, which can be obtained free of charge from The Cambridge Crystallographic Data Centre via www.ccdc.cam.ac.uk/data_request/cif. The structure of these compounds was confirmed in the solid state by single crystal X-ray diffraction technique, which confirmed that the crystal structure contains one molecule in the asymmetric unit. Single crystal structure of **2h** is included as Figure 1 and the crystallographic numbering has been used in the context of the X-ray analysis. Compound **2h** crystallizes in the *P*21/n space group. The molecule is essentially planar with a six-membered intramolecular hydrogen bonding interaction between the oxygen acceptor (O3) and the hydroxyl group with hydrogen bond distance, dO(3)-H(3)^...^O(2) = 1.64 Å. The 3,5-dimethoxyphenyl substituent is also in co-planarity with the 5-acetyl-6-hydroxybenzofuran framework.

In addition to playing essential roles in life processes, enzymes are known to be highly responsive to inhibition by small molecular weight, drug-like molecules and therefore represent attractive targets for drug development and therapy [42]. The planar structure of the *ortho-*hydroxyacetyl 2-arylbenzofuran scaffold would probably facilitate non-covalent interactions with protein residues and, in turn, promote enzyme inhibition. With these considerations in mind, compounds **2a**–**h** were evaluated through in vitro enzymatic assays for inhibitory effect against α-glucosidase, PTP1B and β-secretase activities to correlate between both structural variations and biological activity. Their antioxidant and anti-inflammatory potential have also been evaluated. Structure activity relationship (SAR) was rationalized in terms of substitution pattern of the phenyl substituent on the furan ring.

### 3.2. Biology

#### 3.2.1. Inhibition of α-Glucosidase, PTP1B and β-Secretase and Antioxidant Activity of **2a**–**h**

Compounds **2a**–**h** were evaluated for inhibitory activity against yeast α-glucosidase in vitro using acarbose, a competitive α-glucosidase inhibitor [43] as a reference standard. Acarbose has been found to delay the absorption of carbohydrate from the small intestine and to reduce postprandial hyperglycaemia in patients with T2D than metformin and sulfonylureas [44,45]. The half maximal inhibitory concentration (IC_50_) values were calculated from the dose-dependent curves as the concentrations of inhibitors required to decrease 50% of the enzyme activity (Table 1). No activity against α-glucosidase was observed for the 2-phenyl derivative **2a**. The 3-fluorophenyl substituted derivative **2b** exhibited moderate inhibitory effect against α-glucosidase with an IC_50_ value of 4.65 μM when compared to acarbose (IC_50_ = 0.01 μM). The isomeric derivative **2c** substituted with a 4-fluorophenyl group, on the other hand, displayed significant inhibitory activity with an IC_50_ value of 0.11 μM. The presence of chlorine on the *meta* position of **2d** or *para* position of the phenyl ring of **2e** resulted in loss or significantly reduced activity against this enzyme, respectively. The observed inhibitory effect of compounds **2b** and **2c** can be attributed to the presence of the strongly lipophilic fluorine atom [46,47] and the ability of this atom to form strong hydrogen and halogen bonding interactions with the protein cavity [48]. The difference in activity between **2b** and **2c**, on the other hand, is presumably related to a substitution pattern of the moderately electron delocalizing fluorine atom, which is more pronounced at the *para* position of the benzene ring. The 4-fluorophenyl group of **2c** would probably increase the electron density of the benzofuran scaffold to lead to increased hydrophobic interactions including π-π stacking interactions with protein residues in the enzyme active site. Compound **2f** substituted with a non-polar 4-tolyl group on the 2-position of the furan ring was found to be slightly active against α-glucosidase with an IC_50_ value of 14.07 μM. The presence of the more polar lipophilic 4-methoxyphenyl group on the 2-position of the furan ring, on the other hand, resulted in significant inhibitory effect of **2g** against this enzyme (IC_50_ = 0.56 μM). Significant inhibitory activity against α-glucosidase was also observed for the 3,5-dimethoxyphenyl substituted derivative **2h** with an IC_50_ value of 0.78 μM. Hitherto, the analogous methoxy-substituted 2-arylbenzo[*b*]furans have been found to be inactive against α-glucosidase [49]. The observed activity of **2g** and **2h** is presumably due to the presence of the *ortho-*hydroxyacetyl moiety in analogy with the literature observation for the 2,4-dihydroxy-5-methylacetophenones that have previously been found to exhibit α-glucosidase inhibitory properties [19].

PTP1B knockout studies previously revealed that mice lacking this enzyme exhibit improved sensitivity to insulin and are resistant to a high-fat diet, which induces obesity [50]. Another in vivo study has shown that PTP1B regulates the phosphorylation state of the Y1162/Y1163 site in the activation loop of the IR PTK catalytic domain [51]. As a result, PTP1B-specific inhibitors are expected to enhance insulin sensitivity by prolonging the phosphorylated state of the insulin receptor and act as effective therapeutics for the treatment of T2D and obesity. These observations endorse PTP1B as a key negative regulator of insulin signal transduction and as an attractive target for T2D mellitus treatment [21]. With the aim to develop compounds with potential to simultaneously target α-glucosidase and PTP1B activities, compounds **2a**–**h** were evaluated against PTP1B activity using sodium vanadate (Na_3_VO_4_) as a reference standard. Sodium vanadate is a non-selective inhibitor of protein tyrosine phosphatases (PTPs), which normalizes blood glucose level in diabetes [52]. Compounds **2a**–**h** were found to show enhanced activity profile as compared to sodium vanadate (IC_50_ = 38.4 μM) with IC_50_ values in the range 11.9–31.9 μM. The parent compound **2a** exhibited increased inhibitory effect against PTP1B when compared to the reference standard and its corresponding IC_50_ value is 22.99 μM. The presence of a fluorine or chlorine atom at the *para* position of the phenyl ring, for example, resulted in increased inhibitory effect for compounds **2c** (IC_50_ = 17.8 μM) and **2e** (IC_50_ = 21.5 μM). The isomeric *meta*–substituted derivatives **2b** and **2d**, on the other hand, were found to exhibit slightly lower activity with IC_50_ values of 24.5 μM and 29.3 μM, respectively. The presence of a non-lipophilic methyl group or polar lipophilic methoxy group on the *para* position of the phenyl ring of **2f** and **2g** resulted in relatively less activity for these compounds with IC_50_ values of 26.7 and 31.9 μM, respectively. However, compound **2h** substituted with a 3,5-dimethoxyphenyl group at postion-2 of the furan ring exhibited increased inhibition against PTP1B activity with an IC_50_ value of 11.9 μM. The observed enhanced activity of **2h** is presumably the consequence of increased electron density of the 3,5-dimethoxyphenyl ring, which would facilitate non-covalent interactions of this compound with protein residues in the catalytic and/or allosteric sites of PTP1B. Among the test compounds, the 4-fluorophenyl **2c**, 4-methoxyphenyl **2g** and 3,5-dimethoxyphenyl **2h** substituted derivatives were found to exhibit dual inhibitory effect against α-glucosidase and PTP1B activities.

During diabetes, β-secretase–dependent cleavage of IR is increased and the amount of IR in the liver is reduced [11]. It was observed that infusion of a β-secretase inhibitor leads to partial restoration of the liver IR [11,12]. Aminostyrylbenzofurans, for example, have previously been found to display better inhibitory activities (IC_50_ = 0.07–0.53 μM) than curcumin with an IC_5o_ value of 0.80 μM [12]. This literature observation encouraged us to evaluate compounds **2a**–**h** for potential inhibitory effect against β-secretase activity using quercetin (IC_50_ = 8.76 μM) as a reference standard (Table 1). The test compounds were found to generally exhibit reduced activity against this enzyme when compared to quercetin. Derivatives substituted with a halogenophenyl (**2b**–**2e**) or tolyl group (**2f**) at the 2-position of the furan ring, for example, were found to be inactive against this enzyme when compared to **2a** (IC_50_ = 63.15 μM). Moderate inhibitory effect against β-secretase was, however, observed for the 4-methoxyphenyl and 3,5-dimethoxyphenyl substituted derivatives **2g** and **2h** with IC_50_ values of 27.30 μM and 25.80 μM, respectively.

Oxidative stress is implicated in the early stages and progression of T2D and this stimulated a debate that antioxidants be included as supplements in the standard therapy for diabetes [53]. Research is in progress to develop drugs with dual antidiabetic and antioxidant properties in an effort to ameliorate diabetes complications. In the meantime, the link between anti-diabetic and antioxidant activities of drugs has been confirmed through in vivo experiments and theoretical calculations using protocatechuic acid and glibenclamide as models [54]. The naturally occurring phenolic derivatives such as hydroxytyrosol, resveratrol and curcumin, for example, have been found to exhibit antidiabetic and antioxidant activities in different ways besides other effects like anti-inflammatory and anti-carcinogenic properties [55]. These literature precedents encouraged us to evaluate compounds **2a**–**h** for potential antioxidant properties using the DPPH free-radical scavenging method. The natural antioxidant, ascorbic acid was used as a positive control and the results are expressed as IC_50_ values, that is, the concentration of each sample required or able to scavenge 50% of the DPPH (Table 1). The DPPH radical scavenging assay revealed that most of these compounds possess antioxidant properties. Compound **2a** substituted with a phenyl group on position-2 of the furan ring exhibited significant free radical scavenging activity (IC_50_ = 12.15 μM) when compared to the reference standard (IC_50_ = 10.72 μM). The presence of a fluorine atom on the *meta* position of the phenyl ring of **2b** resulted in significantly reduced antioxidant activity with an IC_50_ value of 46.50 μM. However, its 4-fluorophenyl substituted isomer **2c** exhibited increased antioxidant activity than the reference standard with an IC_50_ value of 9.8 μM. A similar trend was observed for the 3-chlorophenyl substituted derivative **2d** and its 4-chlorophenyl substituted isomer **2e** with IC_50_ values of 15.48 μM and 11.34 μM, respectively. A nonpolar 4-methyl group resulted in significantly reduced anti-radical activity for **2f** (IC_50_ = 26.67 μM) when compared to **2g** substituted at the *para* position of the 2-phenyl ring with a polar and strongly lipophilic methoxy group (IC_50_ = 16.84 μM). Among the test compounds, the highest DPPH scavenger was found to be the *ortho-*hydroxy substituted 5-acetyl-2-(3,5-dimethoxyphenyl)benzo[*b*]furan **2h** with an IC_50_ value of 6.28 μM. This compound also exhibited significant inhibitory effect against α-glucosidase and the highest activity against PTP1B. The analogous methoxy-substituted 2-arylbenzo[*b*]furans have previously been found to exhibit no DPPH radical scavenging activity [49]. The presence of free hydroxyl group, which is capable of donating a hydrogen atom to the odd electron of nitrogen atom in DPPH seems to be essential for the antioxidant activity of compounds **2a**–**h**.

Chronic inflammation is also a common phenomenon present in the background of multiple human disorders including diabetes and the benzofurans are known to exhibit anti-inflammatory properties by targeting of cyclooxygenase-2 activity [15]. Due to the multifactorial origins of diabetes, compounds **2c**, **2g** and **2h**, which exhibited significant activity against α-glucosidase and PTP1B as well as increased free radical scavenging properties in the DPPH assay were, in turn, evaluated for potential anti-inflammatory activity against COX-2 activity.

#### 3.2.2. Inhibitory Assays of **2c**, **2g** and **2h** against COX-2 Activity

Compounds **2c**, **2g** and **2h** were evaluated for potential inhibitory effect against COX-2 using celecoxib as a reference standard and the corresponding results expressed as means of IC_50_ values in µM from duplicate runs are summarized in Table 2 below. Both compounds **2c** and **2g** substituted with moderately electron delocalizing fluorine atom and strongly electron delocalizing methoxy group at the *para* position of the phenyl ring exhibited significant inhibitory effect against COX-2 when compared to celecoxib (IC_50_ = 1.0 µM) with IC_50_ values of 2.8 µM and 2.3 µM, respectively. However, the 3,5-dimethoxyphenyl substituted derivative **2h** which was found to be strongly active against the other targets evaluated in this investigation including β-secretase showed no inhibitory effect against COX-2 activity.

The observed results revealed the *ortho-*hydroxyacetyl substituted benzofuran scaffold to play an important role in evoking the inhibitory activity of these compounds against α-glucosidase and PTP1B as well as their free radical scavenging potential. The difference in the biological activity of these compounds results from the substitution pattern of the 2-phenyl ring. The presence of a halogen atom at the *meta*-position of the 2-phenyl ring seems to be less preferred for biological activity as compared to the isomeric *para* substituted derivatives. However, substitution at the *meta* positions with a strongly lipophilic methoxy group resulted in significant biological activity for **2h**. Kinetic studies were undertaken against α-glucosidase and PTP1B in order to elucidate the plausible molecular mechanism of inhibition of the test compounds.

#### 3.2.3. Kinetic Studies on **2c** and **2h** against α-Glucosidase and PTP1B Activity

**Figure 2:**
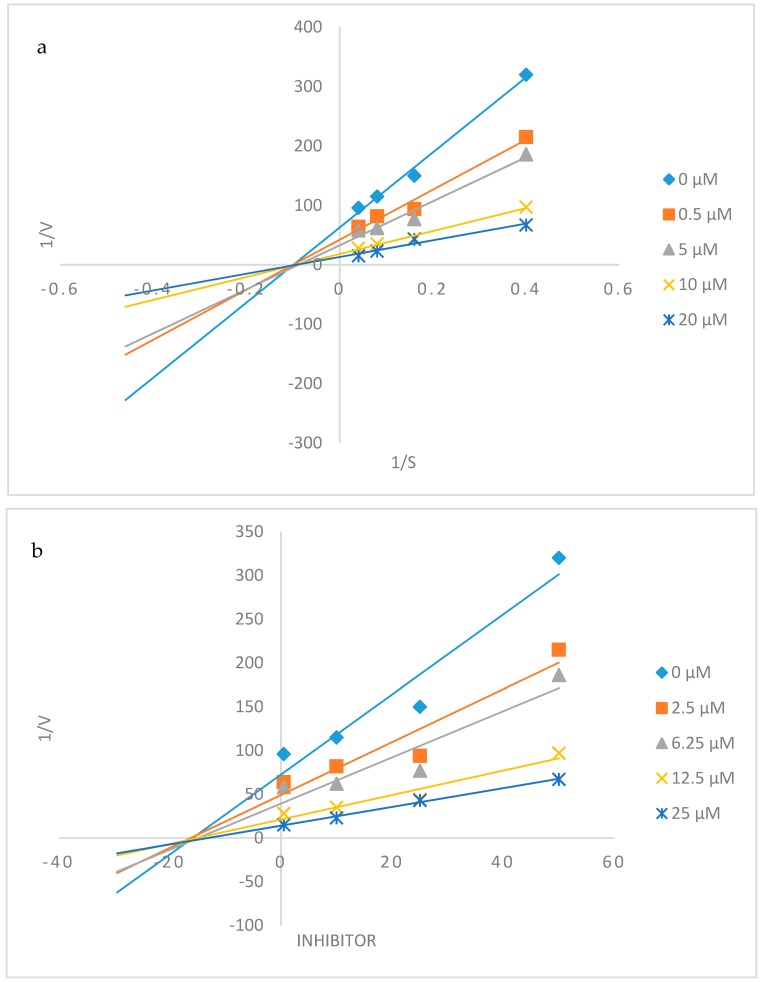
Lineweaver–Burk (**a**) and Dixon (**b**) plots for the inhibition of α-glucosidase by compounds **2c**.

**Figure 3:**
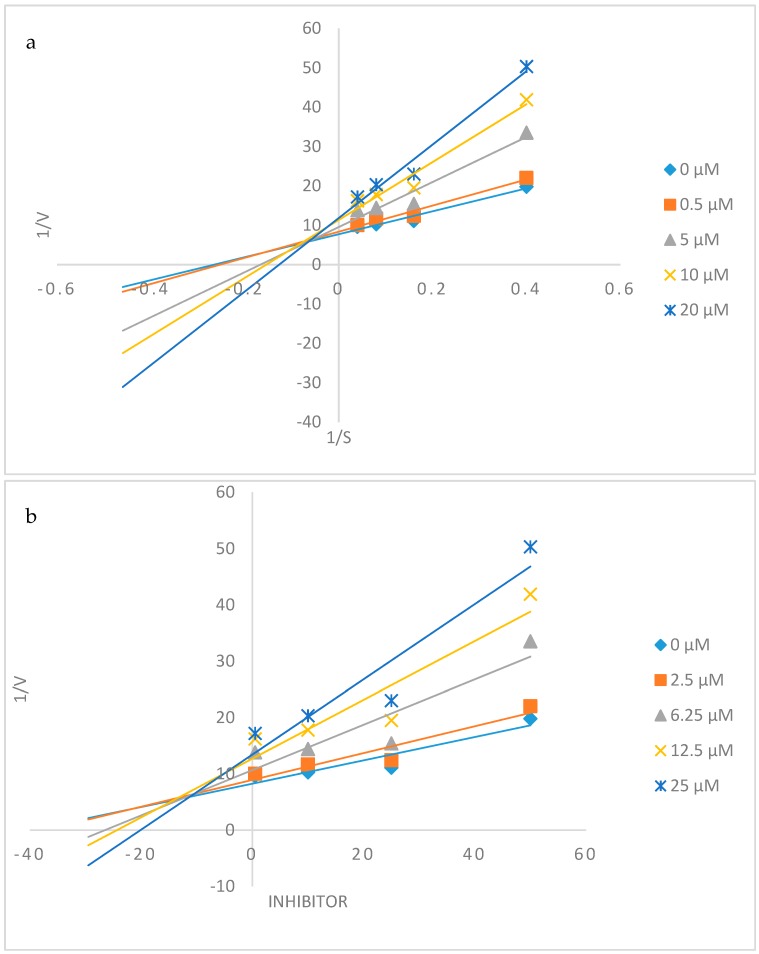
Lineweaver–Burk (**a**) and Dixon (**b**) plots for the inhibition of α-glucosidase by **2h**.

Kinetic analyses of the most active compounds **2c** and **2h** on the inhibition of α-glucosidase and PTP1B were evaluated by constructing the Lineweaver-Burk double reciprocal plots and the Dixon plots at increasing inhibitor and substrate concentrations. The Lineweaver-Burk plot of 1/V versus 1/[S] in the presence of different concentrations of compound **2c** gave a series of straight lines that intersect on the *x*-axis (Figure 2a). The plot shows an unchanged Michaelis constant (K_m_) value and a decrease in the velocity of the reaction (V_max_) values. The Dixon plot intercepts on the axis with the calculated K_i_ value of 15.09 ± 0.03 and this observation is associated with non-competitive mode of inhibition confirming the Lineweaver-Burk data. The Lineweaver-Burk (Figure 3a) and Dixon (Figure 3b) plots for compound **2h** also show set of straight lines intercepting above the *x*-axis. The decreasing K_m_ values (7.87, 6.47, 5.98, 3.95, 3.76) and an unchanged V_max_ (0.19) for this compound is indicative of mixed mode inhibition against the enzyme. While the Dixon plot, with intercepting plot lines above the *x*-axis produce a K_i_ value for **2h** of 5.11 ± 0.42 and indicate a competitive mode of inhibition. It can, therefore, be concluded that this inhibitor behaves in a mixed mode, binding both the active site as well as other allosteric sites on the enzyme.

**Figure 4:**
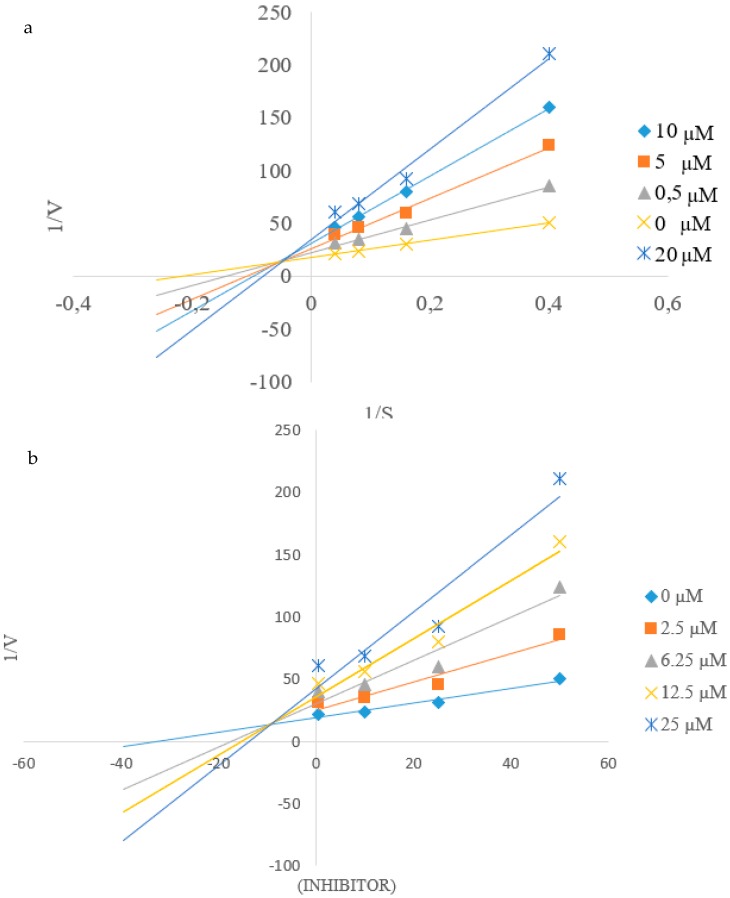
Lineweaver-Burk (**a**) and Dixon (**b**) plots for the inhibition of PTP1B by **2c**.

**Figure 5:**
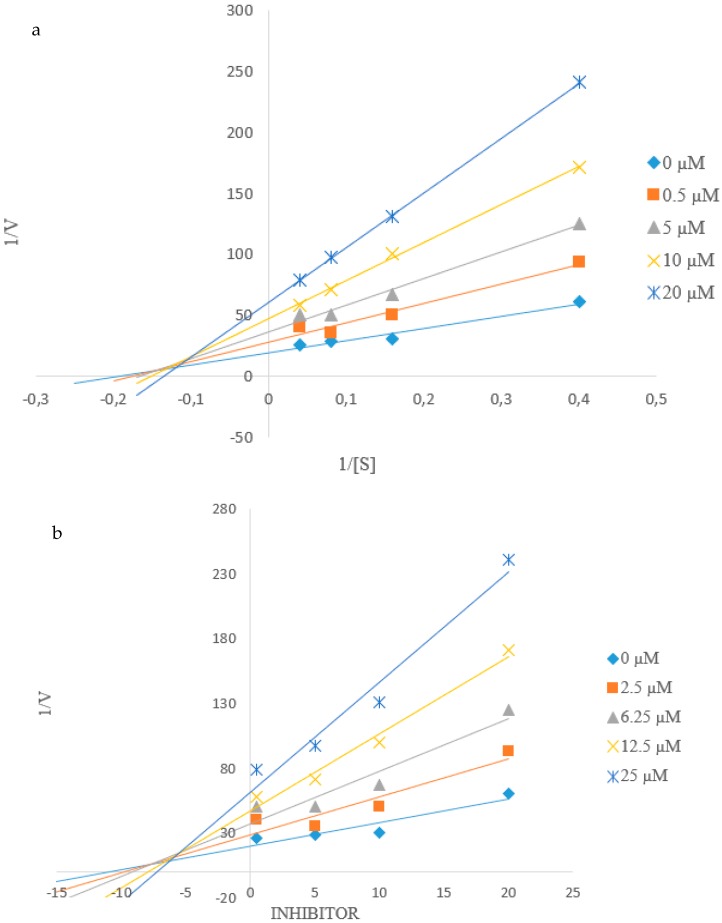
Lineweaver Burke (**a**) and Dixon (**b**) plots for the inhibition of PTP1B by **2h**.

The Lineweaver-Burk (Figure 4a) and Dixon (Figure 4b) plots of compound **2c** for the inhibition of PTP1B displayed series of straight lines all of which intersected just above the *x*-axis. Kinetic analysis of this compound showed a decrease in the velocity of the reaction when the enzyme is fully saturated by substrate, V_max_ (from 47.4 to 6.21), with increasing Michaelis constant values (K_m_ = 0.11–0.52) in the presence of increasing inhibitor concentrations (from 0.5 µM to 20 µM). The observed changes in V_max_ and K_m_ with varying concentration of **2c** suggest a mixed-type mode of inhibition of PTP1B by this compound. Compound **2c** binds to the active site and elsewhere on the enzyme presumably the allosteric site. The Dixon plot of inverse of the velocity of the reaction versus inhibitor concentrations for **2c** produced intersecting lines above the *x*-axis and K_i_ value of 5.2 ± 0.10. The analysis of compound **2h** (Figure 5a) showed that V_max_ decreased (from 36.5 to 7.62) in presence of increasing concentrations of inhibitor (from 0.5 to 20 µM) without changing K_m_ (0.18). This behaviour suggests that this compound exhibited a non-competitive mode of inhibition towards PTP1B, which implies that it binds to the allosteric site independently of substrate. Compound **2h** shares the same affinity for both enzyme and enzyme-substrate complex. An intercepting set of straight lines on the *x*-axis of the Dixon plot of **2h** (Figure 5b) with a K_i_ value of 0.49 ± 0.01 further supports its non-competitive mode of inhibition against PTP1B activity.

### 3.3. Molecular Docking Studies

Automated docking is widely used to predict the geometry of a protein-ligand complex, to rationalize SAR of compounds and for predicting binding affinities of ligands [56]. In the last part of this investigation we employed molecular docking (in silico) to obtain a theoretical/hypothetical model for potential binding modes of compounds **2a**–**h** against α-glucosidase and PTP1B binding sites.

#### 3.3.1. Molecular Docking of **2a**–**h** into α-Glucosidase Binding Site

Comparative modelling was employed to build the three-dimensional structure of *S. cerevisiae* α-glucosidase (maltase) since no crystal structure was reported to-date. Sequence alignment of *S. cerevisiae* α-glucosidase with the available templates, PDB ID 3A47 (*S. cerevisiae* β-glucosidase/isomaltase) and 3AXH (*S. cerevisiae* β-glucosidase/isomaltase) has the sequence identity of 72.06% and 71.89%, respectively (Table S1 in SI). The built model of *S. cerevisiae* α-glucosidase has the average 3D–1D score of > 0.2 and 99% of the residues in the favoured/additional allowed regions (Figure S2 in SI) indicating an acceptable and reasonable predictive three dimensional structure. In addition, the superimposition of the built model of α-glucosidase of *S. cerevisiae* with the available β-glucosidase of *S. cerevisiae* crystal structure (PDB id 3A4A) has the root mean square deviation of 4.16 Å for the Cα backbone atoms (Figure S3 in SI).

**Figure 6:**
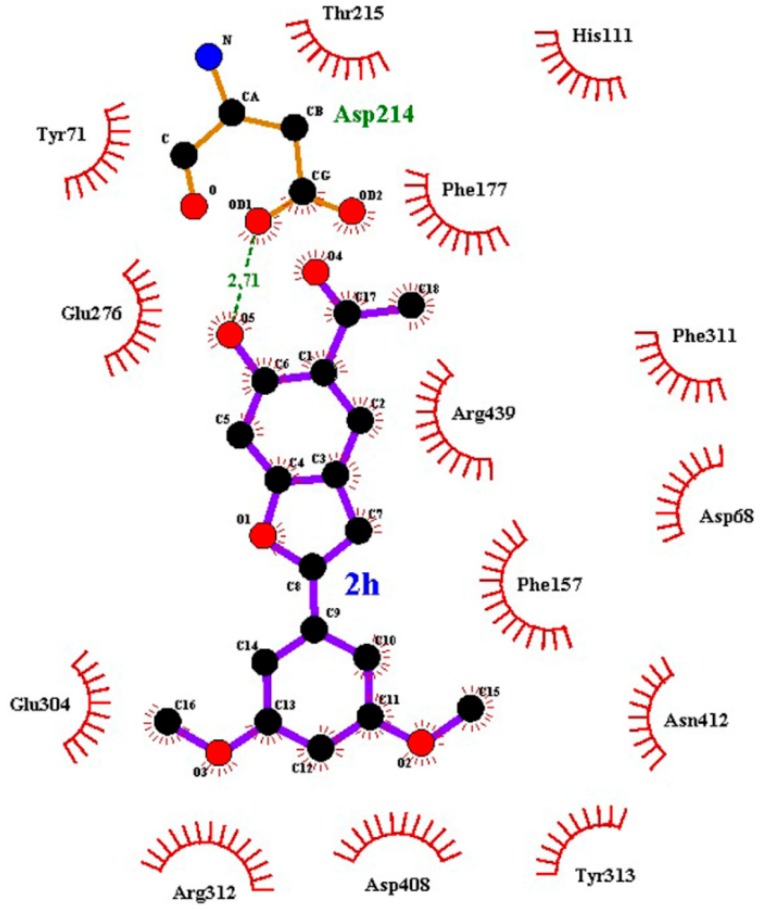
The details interactions of compound **2h** at the binding site of built structure of α-glucosidase.

The control docking of glucose towards the built model of α-glucosidase with root mean square deviation (RMSD) of 0.98 Å with the glucose in β-glucosidase crystal structure (PDB ID 3A4A) showed the appropriateness of the docking parameters. Figure S4 (in SI) shows all compounds docked in similar conformation with the *ortho-*hydroxyacetylbenzofuran framework positioned deep into the binding site, while the phenyl substituent extended nearer to the binding site entrance. The calculated binding energies (kcal/mol) for these compounds are listed in Table 3. The binding energies values obtained through molecular docking do not always correlate well with the biological response observed in the in vitro tests [57]. Nevertheless, the interaction studies between the ligand and protein are useful in rationalizing SAR and for future ligand optimization. Compounds **2g** and **2h** have the most favourable binding free energy (−6.92 kcal/mol) compared with other test compounds in this study (Table 3). With more than 0.5 kcal/mol binding free energy better than the substrate (glucose), compounds **2g** and **2h** are therefore expected to be able to compete with glucose for the binding site, thus able to inhibit α-glucosidase. Further analysis of the interactions of compound **2h** with α-glucosidase showed that the favourable binding affinity is contributed by one hydrogen bond and 14 hydrophobic contacts (Figure 6). The α-glucosidase Phe177, Phe311 and Tyr71 formed stable π-π interactions with compound **2h** benzofuran region while Tyr313 stabilized compound **2h** dimethoxybenzene region.

#### 3.3.2. Molecular Docking of **2a**–**h** into PTP1B Binding Sites

**Figure 7:**
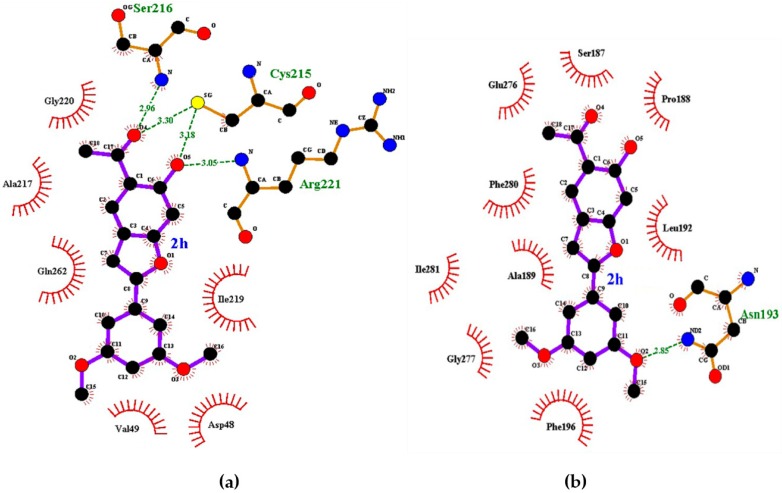
The detailed interactions of **2h** at the catalytic binding site (**a**) and the allosteric binding site of PTB1B (**b**).

The docking of control ligand showed reproducibility of crystal structure where the RMSD values are 1.51 Å and 1.23 Å in PTP1B catalytic (PDB ID: 1NNY) and allosteric (PDB: ID 1T49) site, respectively. A number of small molecules and peptides have shown in vitro and in vivo PTP1B inhibition activity either by binding to the active site or to the allosteric site of this enzyme [48]. Inhibition by targeting the catalytic domain of PTP1B resulted in the inhibition of other protein tyrosine phosphatases family members and therefore significant undesirable off target side effects [58]. It is envisaged that these challenges can be circumvented by targeting the less conserved sites, such as the allosteric site and side pockets present at the borders of the catalytic site [48,58]. Allosteric inhibitors become accommodated into α3-α6-α7 helices destabilizing the network of hydrogen bonds essential for the closure of WPD loop, thereby prohibiting the closure of this loop [58,59]. All the docked test compounds adopted very similar conformation with the benzofuran region positioned deeper into the catalytic site of PTP1B (see Figure S5(a) in SI) while in the allosteric site of PTB1B, all test compounds docked at the benzofuran site of the crystal ligand (refer to Figure S5(b) in SI). The binding affinity of all test compounds are comparable and did not show significant differences in PTP1B catalytic site (Table 4). However, these compounds showed variable binding affinities to the allosteric site of PTP1B, which may suggest that these compounds are bidentate ligands capable of binding to the catalytic site and to the allosteric site as well. The docking poses of **2a**–**h** in the catalytic and allosteric sites are represented below by the docked structure of **2h** in Figure 7a,b, respectively. This compound has the most favourable binding free energy (−7.50 kcal/mol) in PTP1B allosteric site compared with other derivatives (Table 4). Compound **2h** formed a total of four hydrogen bonding and six hydrophobic contacts in PTB1B catalytic site but without any π-π interaction. Hydrogen bonding interactions probably increase the residence time of this compound in the active site of PTP1B and therefore increased activity against this enzyme. Although only one hydrogen bond was detected between compound **2h** and PTP1B allosteric site, the affinity of compound **2h** was favoured by nine hydrophobic contacts (Figure 7b). In addition, the π-π interactions between PTP1B protein residue, Phe196, with dimethoxyphenyl moiety of compound **2h** and Phe280 with its benzofuran scaffold increased the binding affinity of this compound with 59PTP1B allosteric site. The predicted increased hydrophobic interactions of **2h** with protein residues in the allosteric site are consistent with the increased inhibitory effect of this compound. The formation of the hydrogen bond of **2h** with the protein residue Asn193 in the helices of α3–α6 presumably constrains the WPD loop to its open conformation, in turn, inactivates PTP1B. Molecular docking and kinetic data confirm inhibition of **2h** to be more selective for the allosteric site). Literature precedents have confirmed this allosteric site to make such inhibitors highly selective for PTP1B over other PTPs [39,58,59,60].

Although some mismatch was observed between the calculated free energy with the observed IC_50_ data, the interaction study between the ligand and protein could be still useful for future ligand optimization or design.

## 4. Conclusions

The SAR analysis revealed that the *ortho-*hydroxyacetylbenzofuran scaffold plays a significant role in inducing the biological activity of these compounds against α-glucosidase and PTP1B as well as their antioxidant effect. The difference in biological activity results from the nature of substituents and the substitution pattern on the phenyl group at the 2-position of the furan ring. Derivatives **2c**, **2g** and **2h** substituted with the lipophilic 4-fluoro-, 4-methoxy- and 3,5-dimethoxy- group on the phenyl ring, respectively, showed significant inhibitory effect against α-glucosidase and PTP1B activities, more so than the other test compounds in the series. Dual activity against α-glucosidase and PTP1B for compounds **2c**, **2g** and **2h** would probably result in synergistic effects to prevent hyperglycaemia, in turn, effectively improving insulin sensitization without increasing lipid metabolism and causing lipid accumulation in livers. The most active compounds against α-glucosidase, namely, **2c** and **2g** have the potential to retard or prevent glucose absorption into the blood stream and suppress PPHG. Increased lipophilicity of the 3,5-dimethoxyphenyl group, on the other hand, resulted in significant inhibitory activity of **2h** against α-glucosidase and strong inhibitory effect against PTP1B activity. The observed increased activity of **2h** against PTP1B would make it possible for this potential allosteric site inhibitor) to enhance insulin sensitivity and thus act as an effective therapeutic for the treatment of T2D and obesity. The moderate inhibitory effect of **2g** and **2h** against β-secretase, on the other hand, would enhance insulin signalling during diabetes. Dual hyperglycaemic and antioxidant activities of compounds **2c**, **2g** and **2h** make them potential multifunctional anti-diabetic drugs to ameliorate some of the diabetes complications. Cellular-based studies including bioavailability and cell permeability are required to help to clarify the mechanism of action of these compounds in the body and to establish their safety profile as potential multi-target agents against the pathogenesis of T2D.

## Figures and Tables

**Table 1:** PTP1B, α-glucosidase and β-secretase inhibitory activities as well as antioxidant activities of **2a**–**h**.

Compounds	IC_50_ (µM)			
	α-Glucosidase	PTP1B	β-Secretase	DPPH
2a	>100	22.99 ± 0.23	63.15 ± 0.07	12.15 ± 2.10
2b	4.65 ± 0.13	24.50 ± 0.29	>100	46.50 ± 0.24
2c	0.11 ± 0.04	17.75 ± 0.84	>100	9.82 ± 0.86
2d	>100	29.29 ± 0.23	>100	15.48 ± 0.21
2e	64.90 ± 0.05	21.46 ± 0.27	> 100	11.34 ± 1.20
2f	14.05 ± 0.07	26.67 ± 0.30	> 100	26.67 ± 0.62
2g	0.56 ± 0.24	31.88 ± 0.46	27.30 ± 1.10	16.84 ± 0.38
2h	0.78 ± 0.31	11.90 ± 0.35	25.80 ± 0.08	6.28 ± 0.33
Acarbose	0.01 ± 0.02	-	-	-
Na_2_VO_4_	-	38.42 ± 0.28	-	-
Quercetin	-	-	8.76 ± 0.10	-
Ascorbic acid	-	-	-	10.72 ± 0.42

PTP1B: protein tyrosine phosphatase 1B; IC_50_; half maximal inhibitory concentration; DPPH: 2,2-diphenyl-1-picrylhydrazyl

**Table 2:** Activity of **2c**, **2g** and **2h** against COX-2.

Compounds	COX-2 (IC_50_ µM)
**2c**	2.81 ± 0.70
**2g**	2.33 ± 1.52
**2h**	99.55 ± 2.60
Celecoxib	1.02 ± 1.53

COX-2: cyclooxygenase-2.

**Table 3:** Estimated binding free energy (kcal/mol) calculated from docking simulation for control docking and test compound **2a–2h** with the built structure of α-glucosidase.

Compound	α-Glucosidase
Control	−5.94
**2a**	−6.49
**2b**	−6.43
**2c**	−6.27
**2d**	−6.58
**2e**	−6.11
**2f**	−6.70
**2g**	−6.92
**2h**	−6.92

**Table 4:** Estimated binding free energy (kcal/mol) calculated from docking simulation for control docking and compounds **2a**–**2h** with PTB1B.

Compound	PTB1B	
	Catalytic Site	Allosteric Site
Control	−7.81	−11.20
**2a**	−5.45	−6.85
**2b**	−5.43	−6.85
**2c**	−5.42	−6.82
**2d**	−5.60	−7.09
**2e**	−5.64	−7.10
**2f**	−5.58	−7.05
**2g**	−5.35	−6.82
**2h**	−5.43	−7.50

## Supplementary Materials

The following are available online at https://www.mdpi.com/2218-273X/10/3/418/s1, Figure S1: ^1^H-NMR and ^13^C-NMR spectra of compounds **2a**–**h**, Table S1**: ** The sequence alignment of *Saccharomyces cerevisiae* α-glucosidase (maltase) with templates, PDB ID 3A47 and 3AXH, Figure S2: The Ramachandran plot of the comparative model of *S. cerevisiae* α-glucosidase, Figure S3: The superimposition of the comparative model of *S. cerevisiae* α-glucosidase with the crystal structure of β-glucosidase of *S. cerevisiae* (PDB ID 3A4A), Figure S4: The docked conformation of the test compounds at the binding site of α-glucosidase built model (PDB ID 3A4A), and Figure S5: The docked conformation of the test compounds at (**a**) the catalytic binding site of PTB1B, and (**b**) the allosteric binding site of PTB1B.
