# Supplementary material for: Synthesis, In Vitro Evaluation and Molecular Docking of the 5-Acetyl-2-aryl-6-hydroxybenzo[b]furans against Multiple Targets Linked to Type 2 Diabetes
Source: Biomolecules. 2020 Mar 7;10(3):418. doi: 10.3390/biom10030418 (PMC7175131; doi:10.3390/biom10030418)
Supplement: Supplementary file 1 [file biomolecules-10-00418-s001.pdf]

Supplementary Information

Figure S1: ^{1}H-NMR and ^{13}C-NMR spectra of compounds **2a–h**.

Table S1. The sequence alignment of *Saccharomyces cerevisiae* α-glucosidase (maltase) with templates, PDB ID 3A47 and 3AXH.

Figure S2: The Ramachandran plot of the comparative model of *S. cerevisiae* α-glucosidase.

Figure S3: The superimposition of the comparative model of *S. cerevisiae* α-glucosidase with the crystal structure of β-glucosidase of *S. cerevisiae* (PDB id 3A4A).

Figure S4: The docked conformation of the test compounds at the binding site of α-glucosidase built model (PDB id 3A4A).

Figure S5: The docked conformation of the test compounds at (**a**) the catalytic binding site of PTB1B, and (**b**) the allosteric binding site of PTB1B.

Figure S1: ^{1}H-NMR and ^{13}C-NMR spectra of compounds **2a–h.**

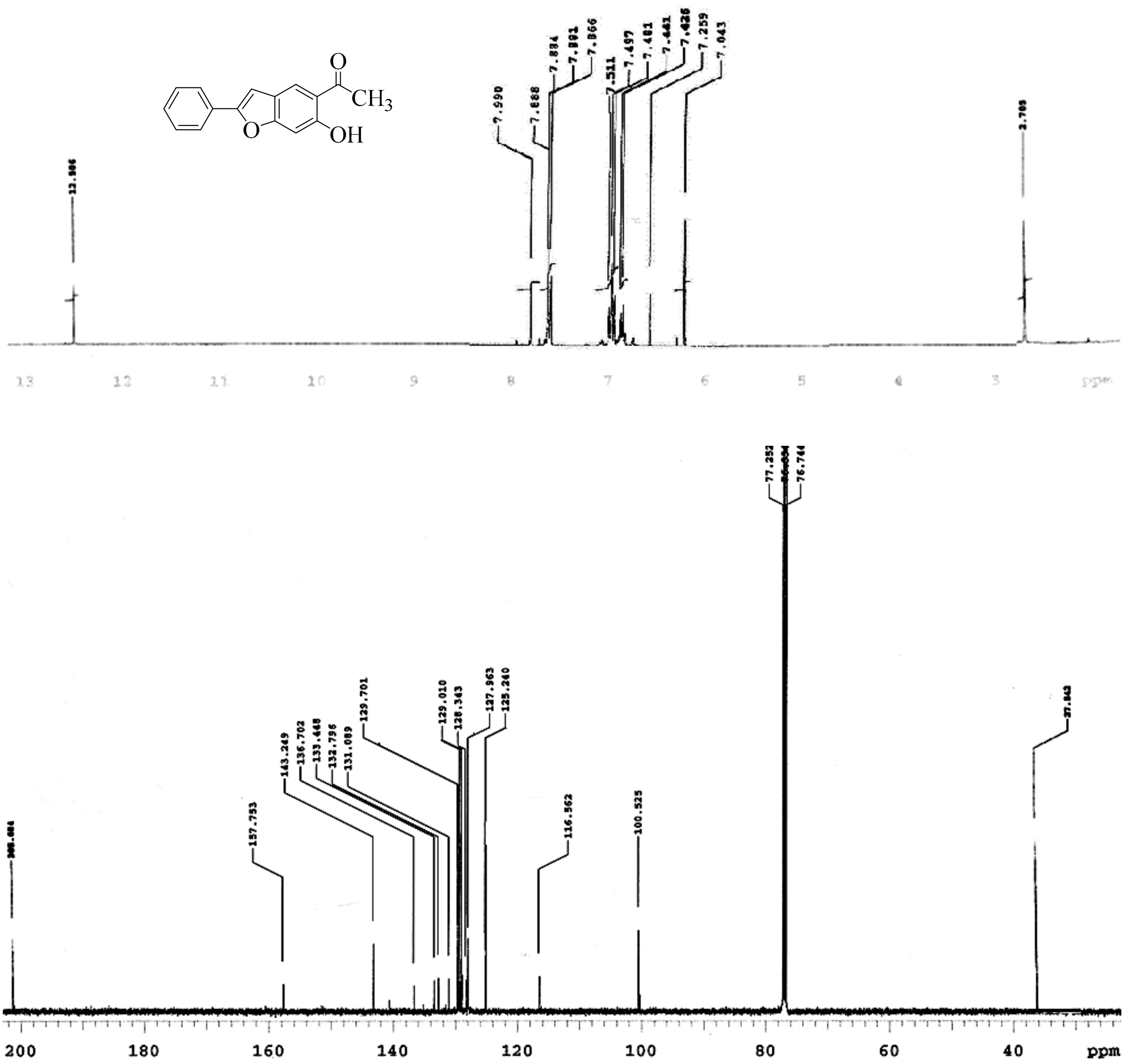

Figure S1.1: ^{1}H-NMR and ^{13}C-NMR spectra of **2a** in $CDCl_3$ at 500 MHz and 125 MHz, respectively.

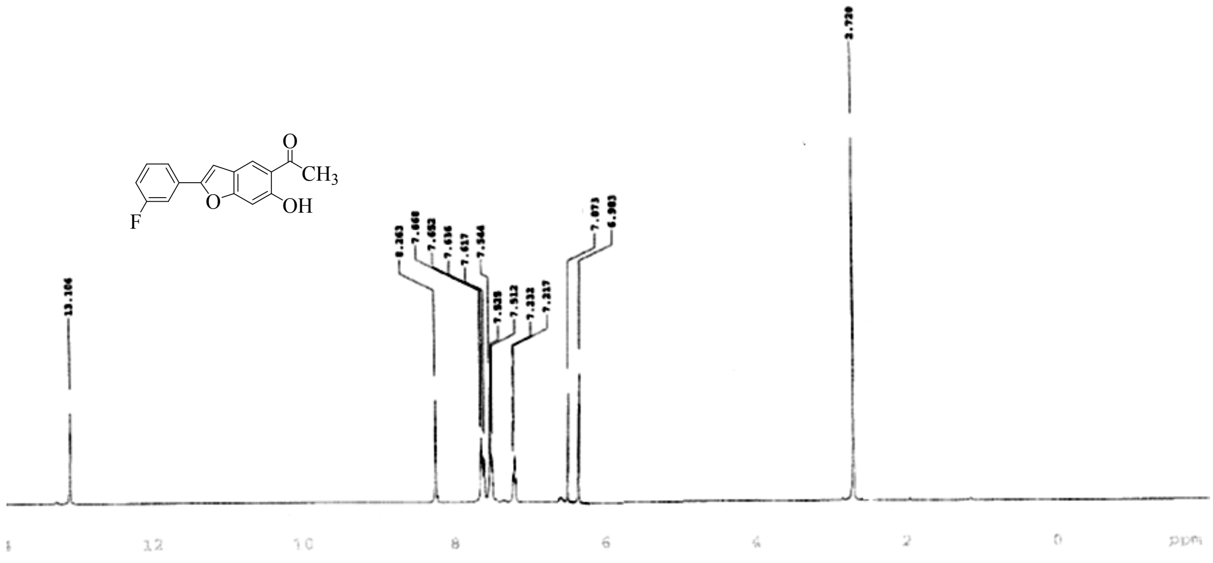

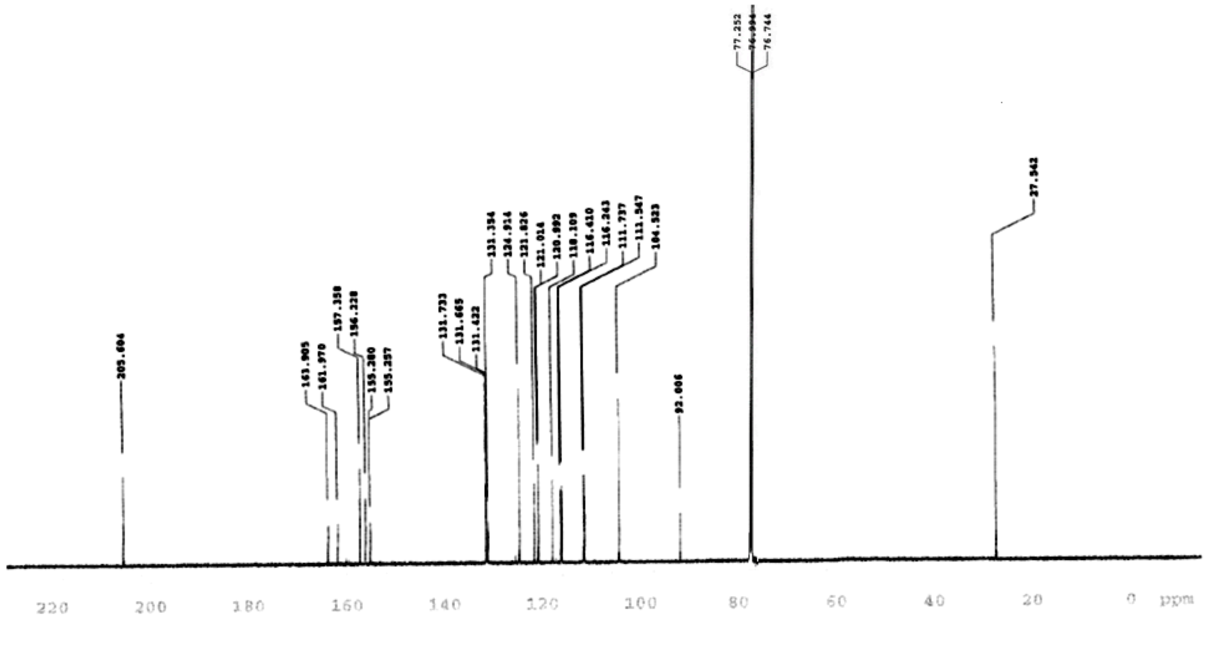

Figure S1.2: ^{1}H-NMR and ^{13}C-NMR spectra of **2b** in $CDCl_3$ at 500 MHz and 125 MHz, respectively.

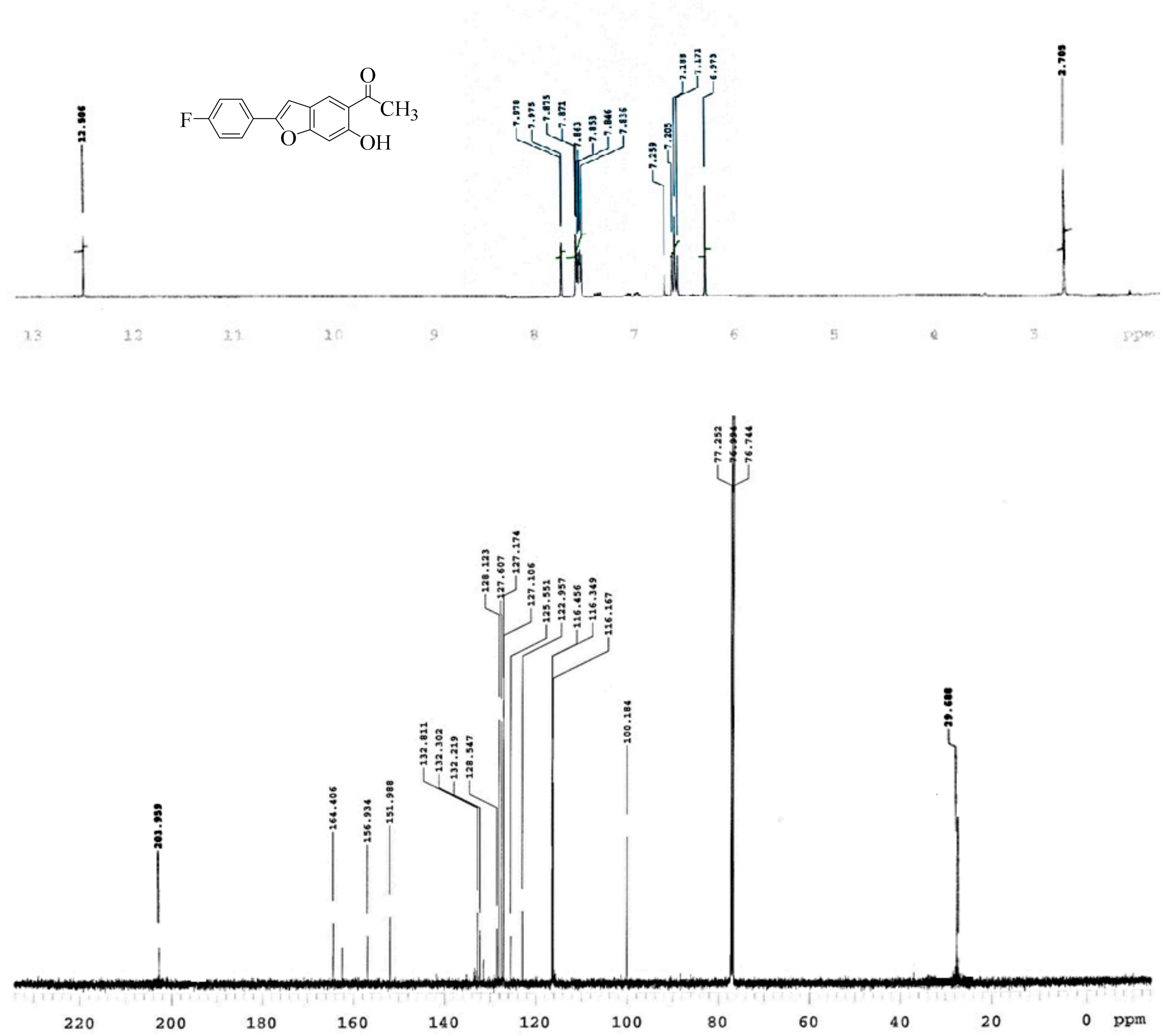

Figure S1.3: ^{1}H-NMR and ^{13}C-NMR spectra of **2c** in $CDCl_3$ at 500 MHz and 125 MHz, respectively.

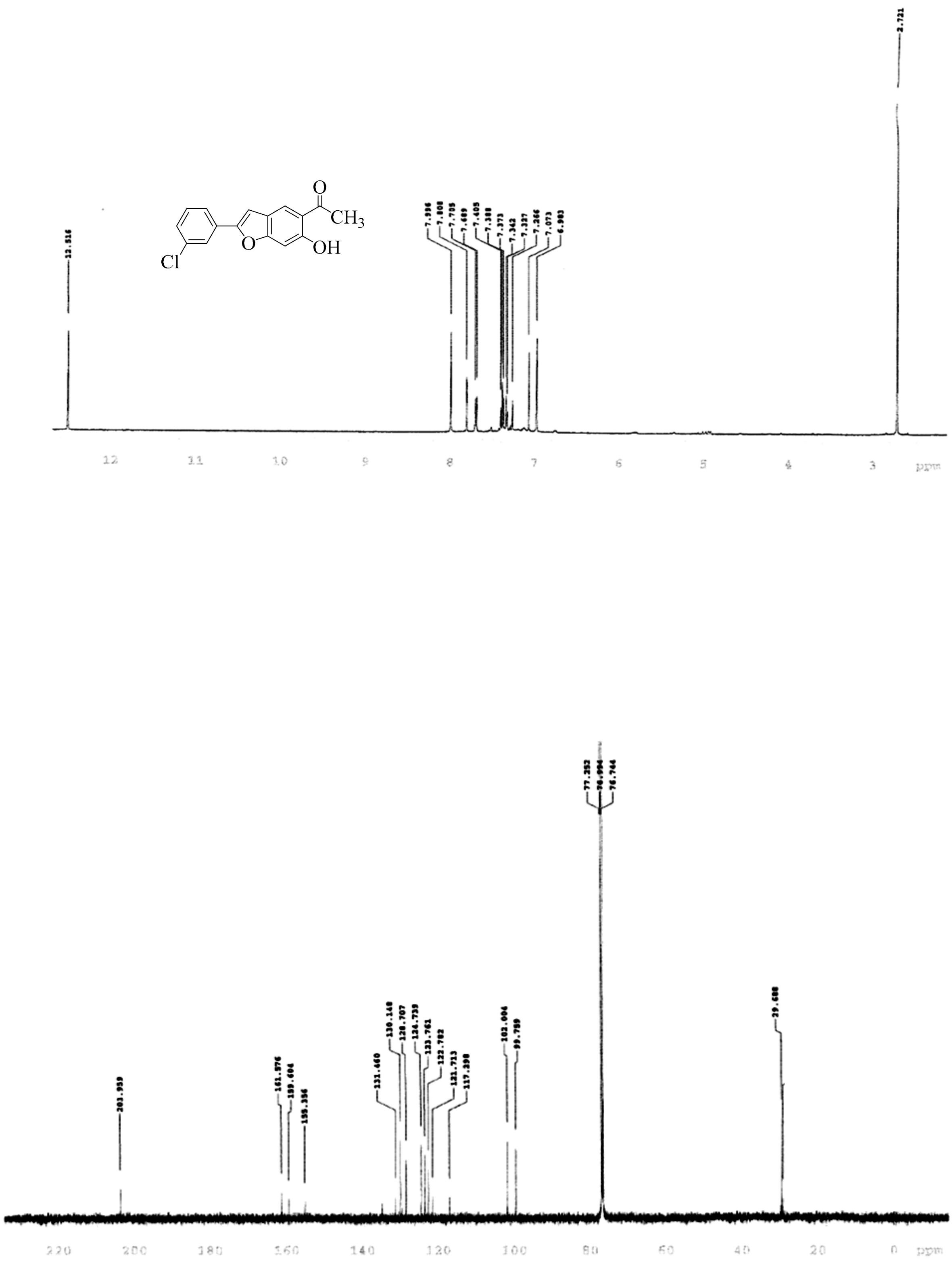

Figure S1.4: ^{1}H-NMR and ^{13}C-NMR spectra of **2d** in $CDCl_3$ at 500 MHz and 125 MHz, respectively.

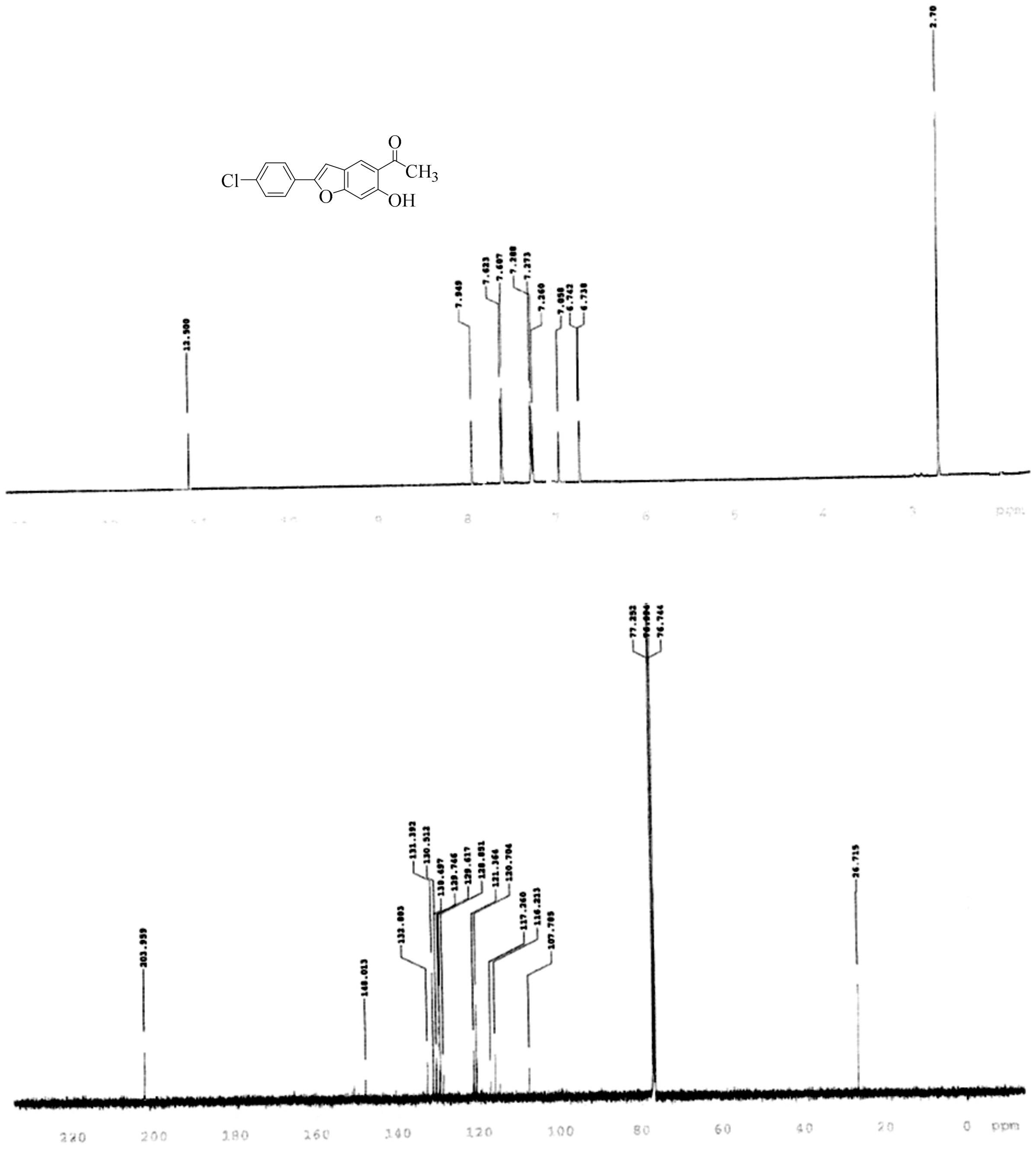

Figure S1.5: ^{1}H-NMR and ^{13}C-NMR spectra of **2e** in $CDCl_3$ at 500 MHz and 125 MHz, respectively.

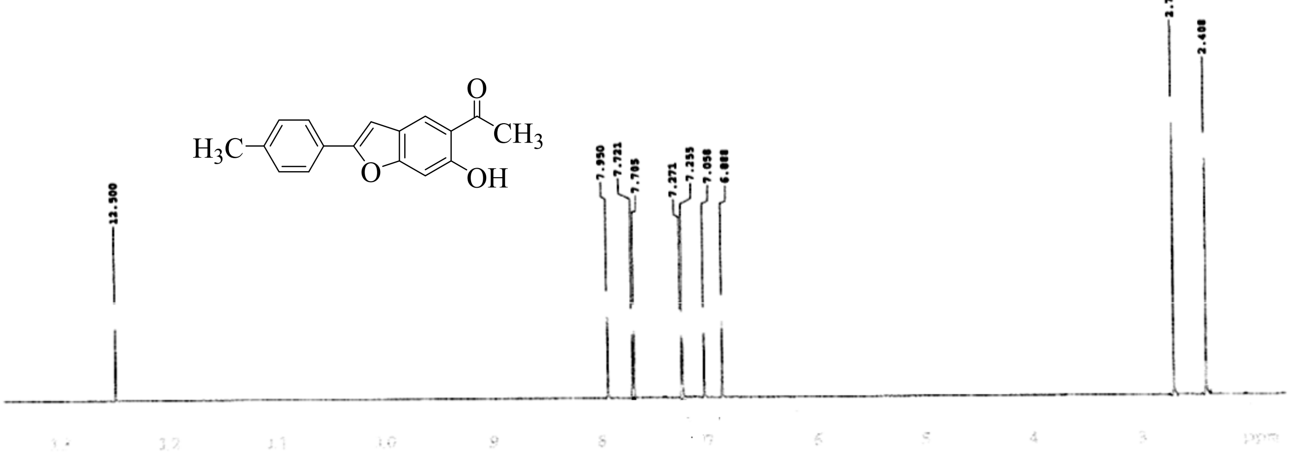

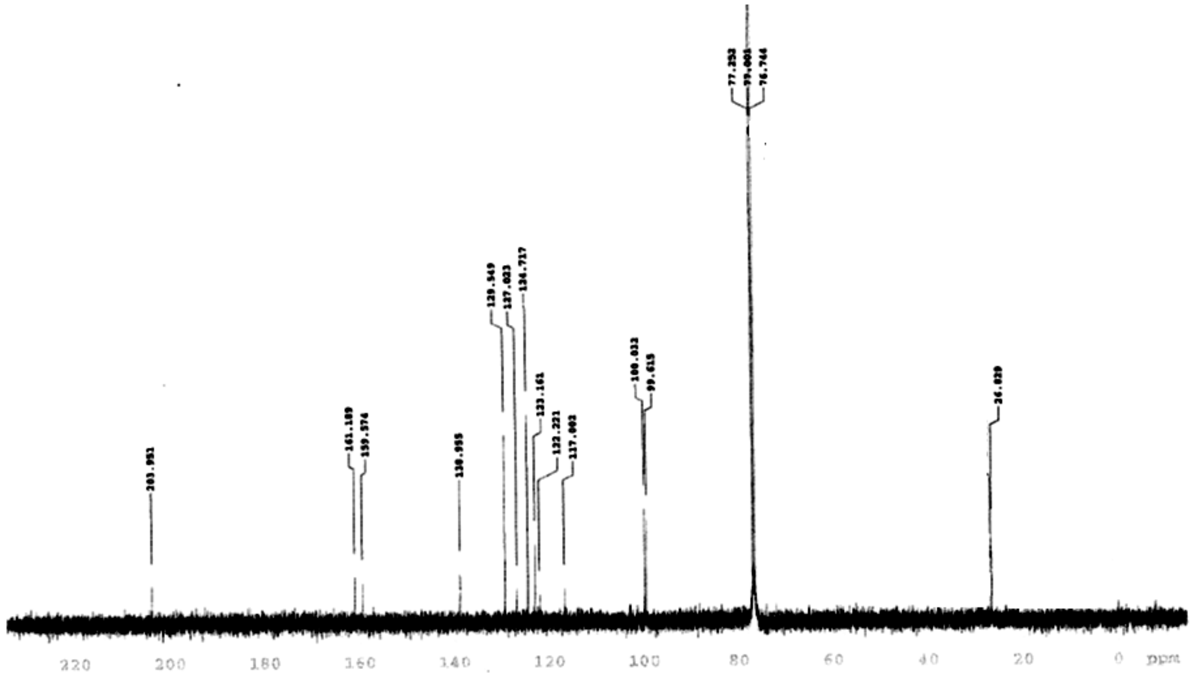

Figure S1.6: ^{1}H-NMR and ^{13}C-NMR spectra of **2f** in $CDCl_3$ at 500 MHz and 125 MHz, respectively.

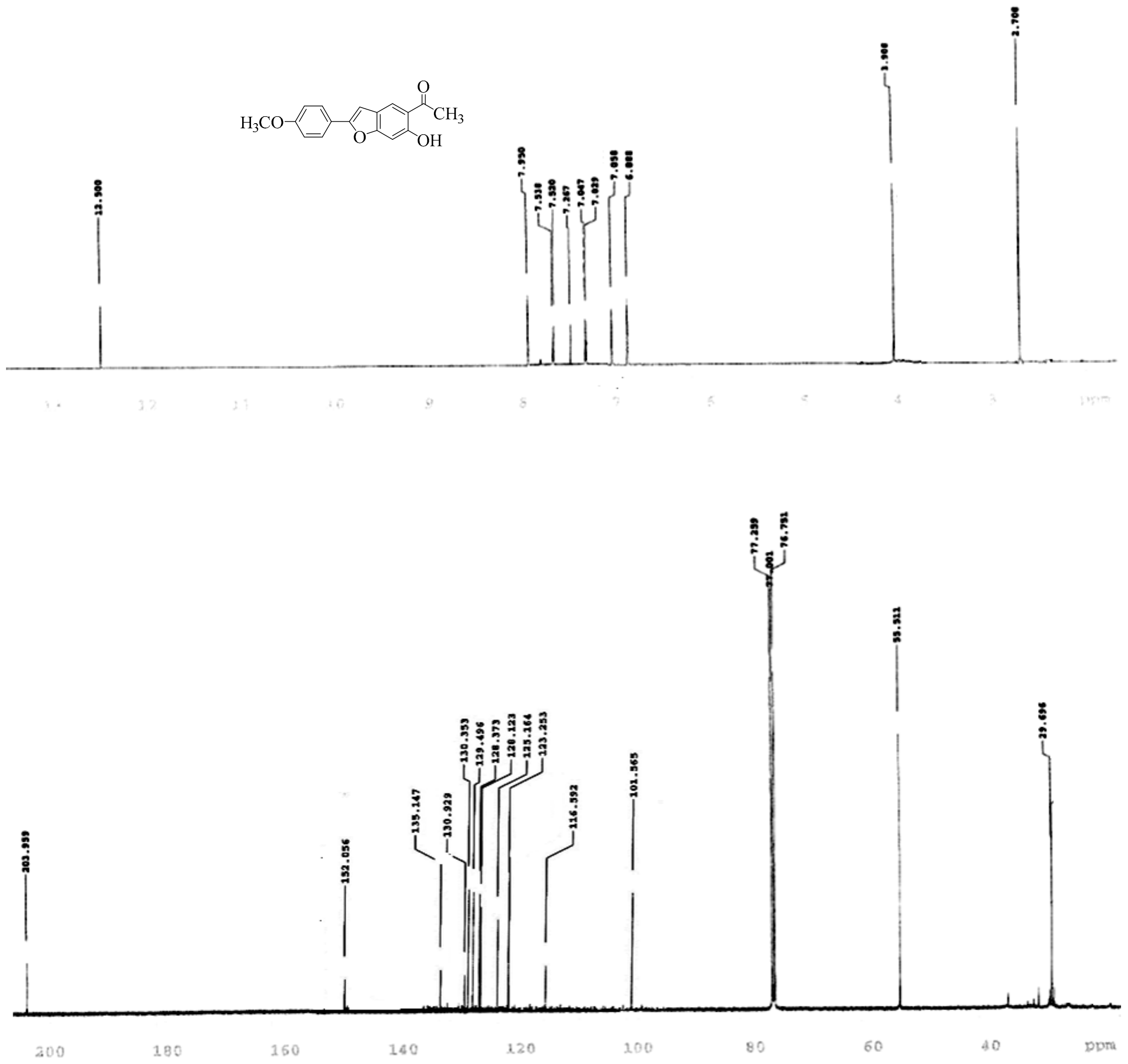

Figure S1.7: ^{1}H-NMR and ^{13}C-NMR spectra of **2g** in $CDCl_3$ at 500 MHz and 125 MHz, respectively.

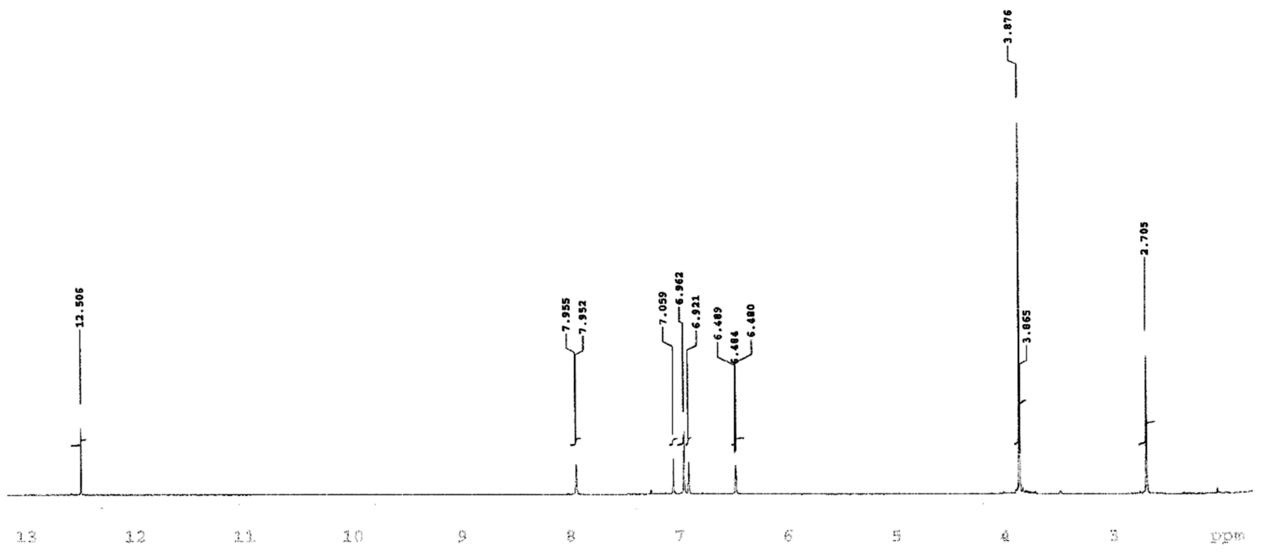

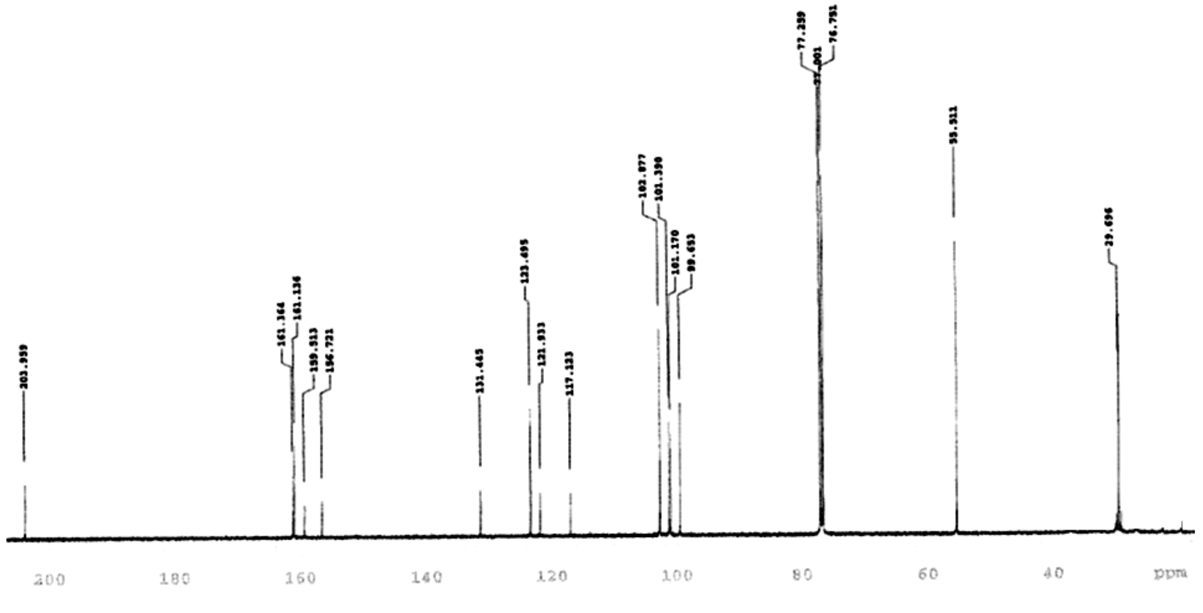

Figure S1.8: ^{1}H-NMR and ^{13}C-NMR spectra of **2h** in $CDCl_3$ at 500 MHz and 125 MHz, respectively.

Table S1. The sequence alignment of *Saccharomyces cerevisiae* α-glucosidase (maltase) with templates, PDB ID 3A47 and 3AXH.

```
                        10        20        30        40        50        60        70        80
3A47A           S--SAHPETEPKWWKEATFYQIYPASFKDSNDDGWGDMKGIASKLEYIKELGADAIWISPFYDSPQDDMGYDIANYEKVW
3AXHA           S--SAHPETEPKWWKEATFYQIYPASFKDSNDDGWGDMKGIASKLEYIKELGADAIWISPFYDSPQDDMGYDIANYEKVW
α-glucosidase   MTISDHPETEPKWWKEATIYQIYPASFKDSNNDGWGDLKGITSKLQYIKDLGVDAIWVCPFYDSPQQDMGYDISNYEKVW
                   * ************* ************ ***** *** *** *** ** ****  ******* ****** ******

                        90       100       110       120       130       140       150       160
3A47A           PTYGTNEDCFALIEKTHKLGMKFITDLVINHCSSEHEWFKESRSSKTNPKRDWFFWRPPKGYDAEGKPIPPNNWKSYFGG
3AXHA           PTYGTNEDCFALIEKTHKLGMKFITDLVINHCSSEHEWFKESRSSKTNPKRDWFFWRPPKGYDAEGKPIPPNNWKSYFGG
α-glucosidase   PTYGTNEDCFELIDKTHKLGMKFITDLVINHCSTEHEWFKESRSSKTNPKRDWFFWRPPKGYDAEGKPIPPNNWKSFFGG
                ********** ** ******************* ****************************************** ***

                       170       180       190       200       210       220       230       240
3A47A           SAWTFDEKTQEFYLRLFCSTQPDLNWENEDCRKAIYESAVGYWLDHGVDGFRIDVGSLYSKVVGLPDAPVVDKNSTWQSS
3AXHA           SAWTFDEKTQEFYLRLFCSTQPDLNWENEDCRKAIYESAVGYWLDHGVDGFRIDVGSLYSKVVGLPDAPVVDKNSTWQSS
α-glucosidase   SAWTFDETTNEFYLRLFASRQVDLNWENEDCRRAIFESAVGFWLDHGVDGFRIDTAGLYSKRPGLPDSPIFDKTSKLQHP
                ******* * ******* * * ********** ** ***** ************   ****  **** *  ** *  *

                       250       260       270       280       290       300       310       320
3A47A           DPYTLNGPRIHEFHQEMNQFIRNRVKDGREIMTVGEMQHASDETKRLYTSASRHELSELFNFSHTDVGTSPLFRYNLVPF
3AXHA           DPYTLNGPRIHEFHQEMNQFIRNRVKDGREIMTVGAMQHASDETKRLYTSASRHELSELFNFSHTDVGTSPLFRYNLVPF
α-glucosidase   NWGSHNGPRIHEYHQELHRFMKNRVKDGREIMTVGEVAHGSD--NALYTSAARYEVSEVFSFTHVEVGTSPFFRYNIVPF
                     ******* ***   *  *************   * **    ***** * * ** * * *  ***** **** ***

                       330       340       350       360       370       380       390       400
3A47A           ELKDWKIALAELFRYINGTDCWSTIYLENHDQPRSITRFGDDSPKNRVISGKLLSVLLSALTGTLYVYQGQELGQINFKN
3AXHA           ELKDWKIALAELFRYINGTDCWSTIYLENHDQPRSITRFGDDSPKNRVISGKLLSVLLSALTGTLYVYQGQELGQINFKN
α-glucosidase   TLKQWKEAIASNFLFINGTDSWATTYIENHDQARSITRFADDSPKYRKISGKLLTLLECSLTGTLYVYQGQEIGQINFKE
                 ** ** * *  *  ***** * * * ***** ****** ***** * ******  *   ************ ******

                       410       420       430       440       450       460       470       480
3A47A           WPVEKYEDVEIRNNYNAIKEEHGENSEEMKKFLEAIALISRDHARTPMQWSREEPNAGFSGPSAKPWFYLNDSFREGINV
3AXHA           WPVEKYEDVEIRNNYNAIKEEHGENSEEMKKFLEAIALISRDHARTPMQWSREEPNAGFSGPSAKPWFYLNDSFREGINV
α-glucosidase   WPIEKYEDVDVKNNYEIIKKSFGKNSKEMKDFFKGIALLSRDHSRTPMPWTKDKPNAGFTGPDVKPWFLLNESFEQGINV
                ** ******   ***  **   * ** *** *   *** **** **** *    ***** **  ****** **  *****

                       490       500       510       520       530       540       550       560
3A47A           EDEIKDPNSVLNFWKEALKFRKAHKDITVYGYDFEFIDLDNKKLFSFTKKYNNKTLFAALNFSSDATDFKIPNDDSSFKL
3AXHA           EDEIKDPNSVLNFWKEALKFRKAHKDITVYGYDFEFIDLDNKKLFSFTKKYNNKTLFAALNFSSDATDFKIPNDDSSFKL
α-glucosidase   EQESRDDDSVLNFWKRALQARKKYKELMIYGYDFQFIDLDSDQIFSFTKEYEDKTLFAALNFSGEEIEFSLPREGASLSF
                * *  *  ******* **  **       ***** *****    ***** *  **********     *  *    *

                       570       580
3A47A           EFGNYPKKEVDASSRTLKPWEGRIYISE
3AXHA           EFGNYPKKEVDASSRTLKPWEGRIYISE
α-glucosidase   ILGNYD--DTDVSSRVLKPWEGRIYLVK
                  ***     * *** *********
```

* The conserved residues.

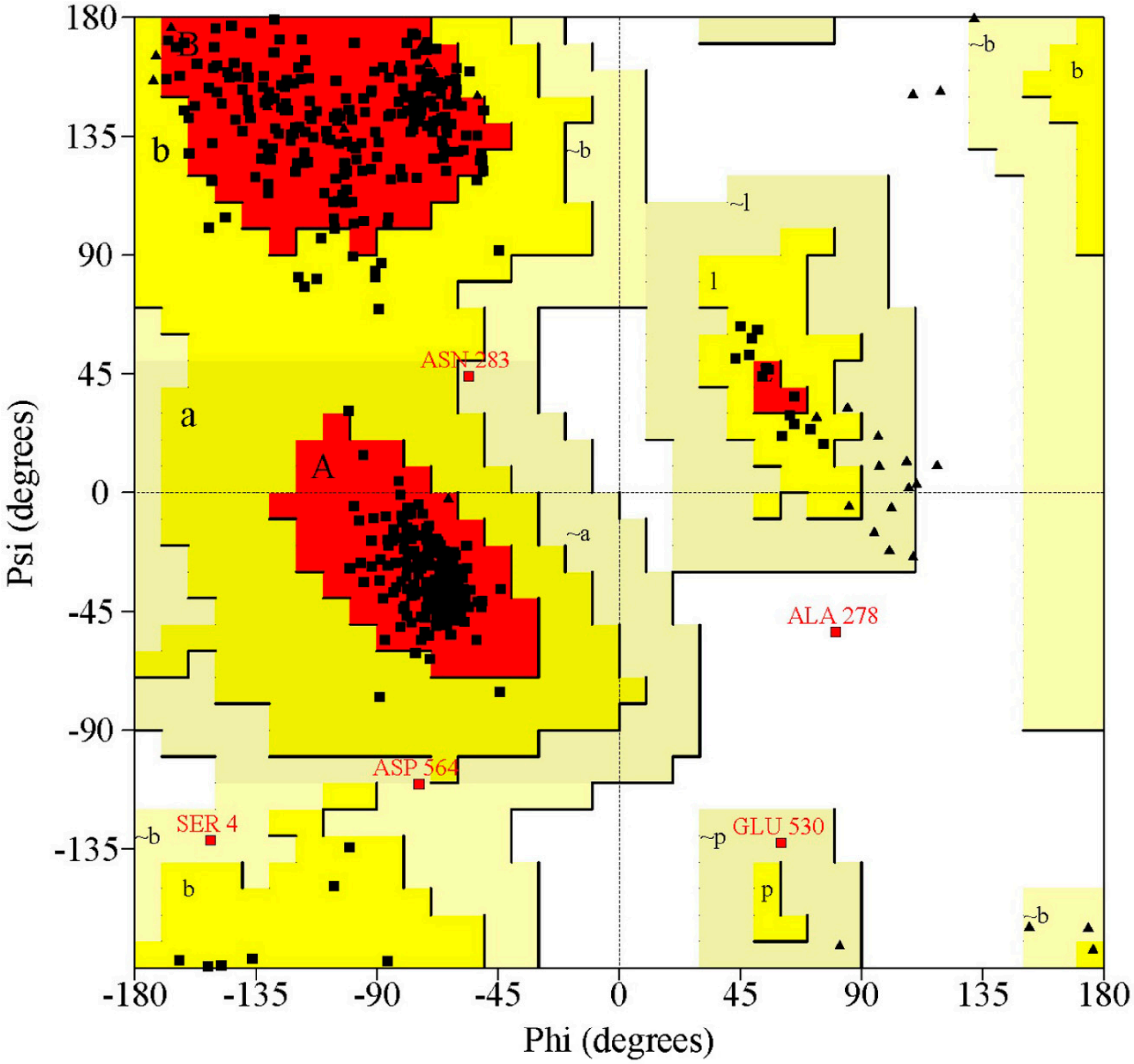

Plot statistics

Residues in most favoured regions [A,B,L]	475	91.9%
Residues in additional allowed regions [a,b,l,p]	37	7.2%
Residues in generously allowed regions [~a,~b,~l,~p]	4	0.8%
Residues in disallowed regions	1	0.2%
	----	------
Number of non-glycine and non-proline residues	517	100.0%
Number of end-residues (excl. Gly and Pro)	2	
Number of glycine residues (shown as triangles)	37	
Number of proline residues	28	

Total number of residues	584	

Based on an analysis of 118 structures of resolution of at least 2.0 Angstroms and R-factor no greater than 20%, a good quality model would be expected to have over 90% in the most favoured regions.

Figure S2. The Ramachandran plot of the comparative model of *S. cerevisiae* α-glucosidase.

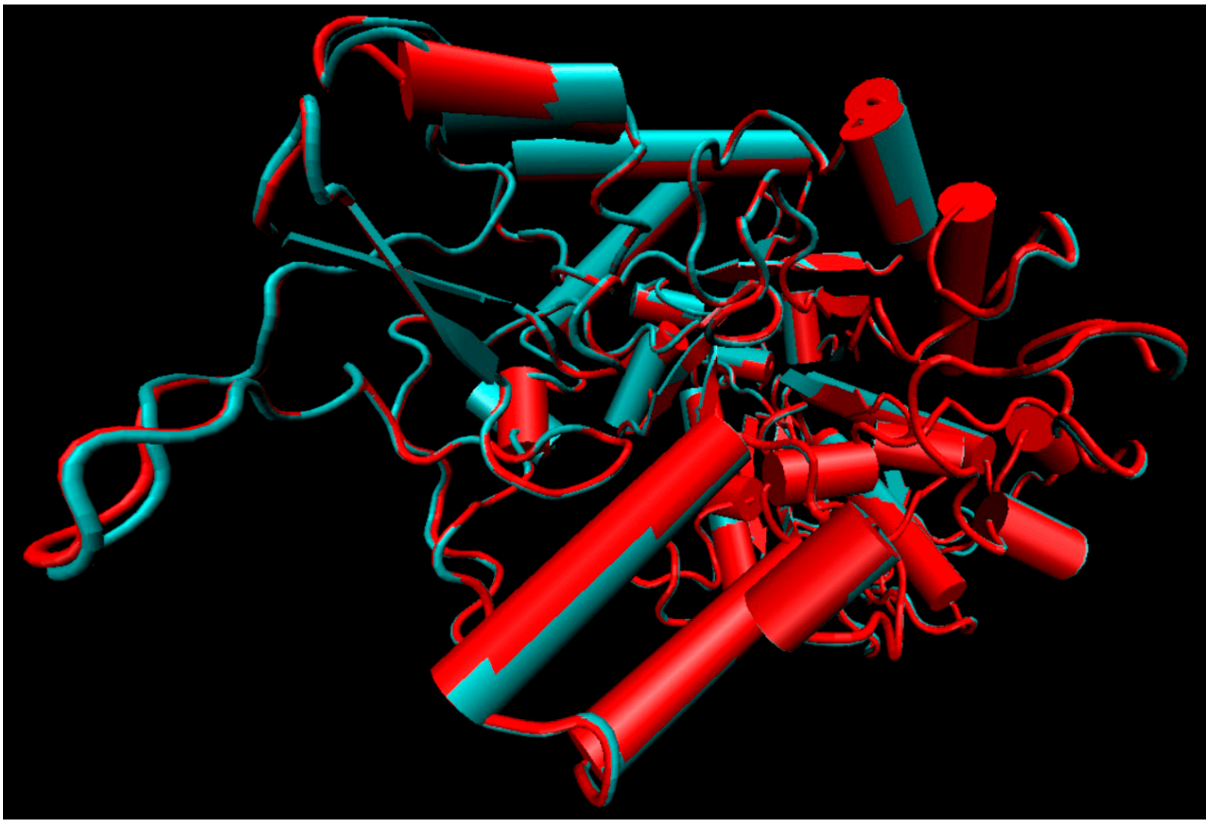

Figure S3. The superimposition of the comparative model of *S. cerevisiae* α-glucosidase (red ribbon presentation) with the crystal structure of β-glucosidase of *S. cerevisiae* (PDB id 3A4A; cyan ribbon presentation). The Cα backbone atoms has the RMSD of 4.16 Å between the two proteins.

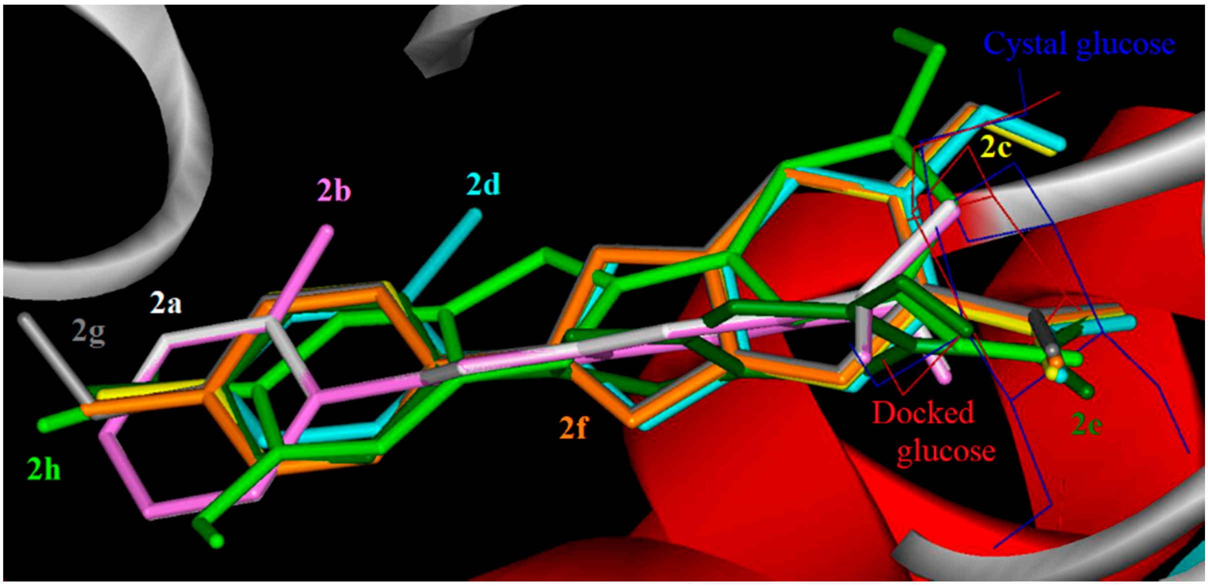

Figure S4: The docked conformation of the test compounds (stick presentation) at the binding site of α-glucosidase built model (ribbon presentation, the "crystal glucose" is from the ligand of PDB id 3A4A). The RMSA value between the docked glucose and crystal glucose is 0.98 Å.

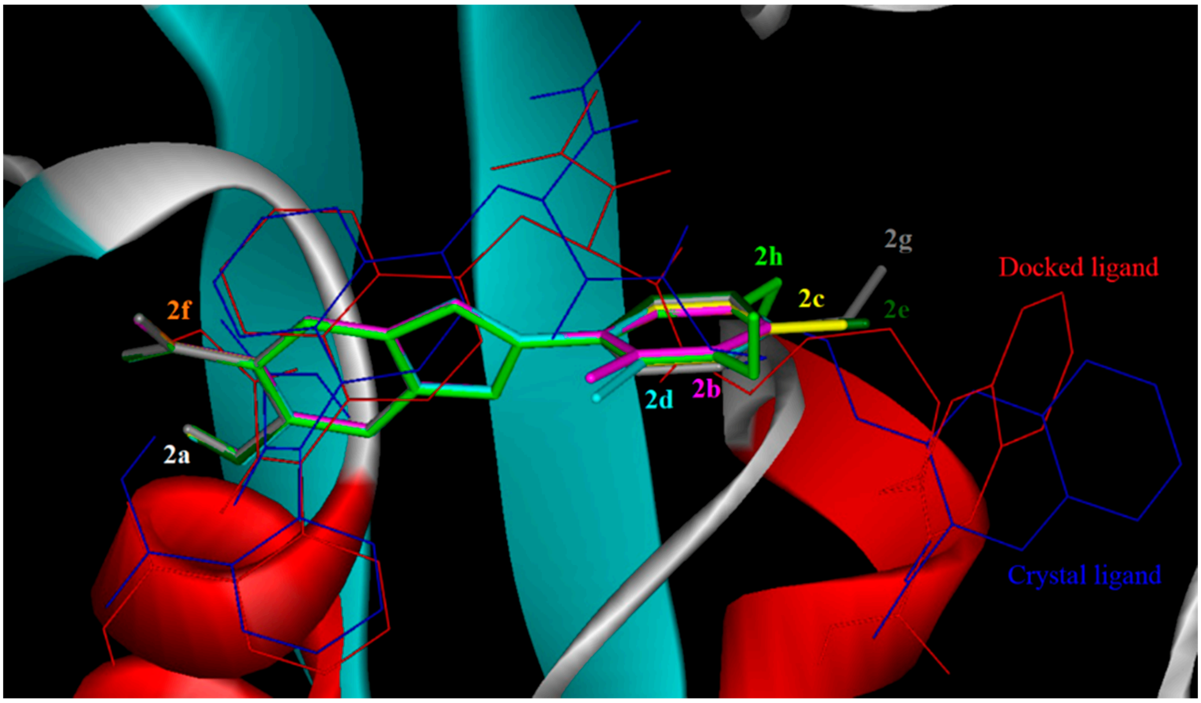

Figure S5 (a)

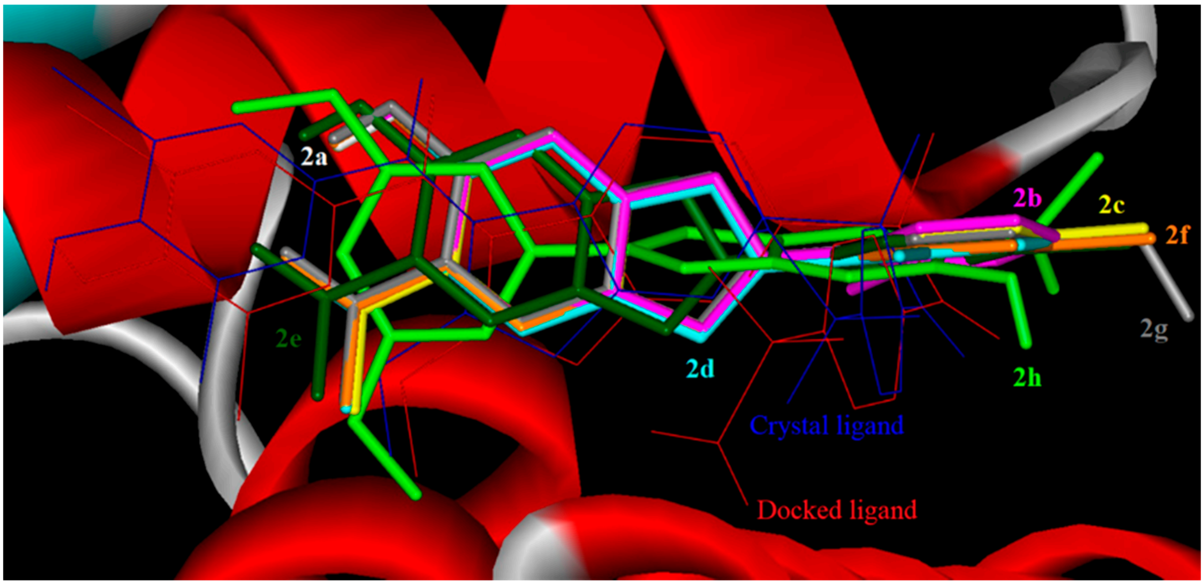

Figure S5 (b)

Figure S5: The docked conformation of the test compounds (stick presentation) at **(a)** the catalytic binding site of PTB1B (ribbon presentation) and, **(b)** the allosteric binding site of PTB1B (ribbon presentation).
